# Role of the PI3K/AKT signaling pathway in the cellular response to Tumor Treating Fields (TTFields)

**DOI:** 10.1038/s41419-025-07546-8

**Published:** 2025-03-27

**Authors:** Anat Klein-Goldberg, Tali Voloshin, Efrat Zemer Tov, Rom Paz, Lina Somri-Gannam, Alexandra Volodin, Lilach Koren, Lena Lifshitz, Aviv Meir, Ayelet Shabtay-Orbach, Roni Blatt, Shay Cahal, Catherine Tempel-Brami, Kerem Wainer-Katsir, Tal Kan, Bella Koltun, Boris Brant, Yiftah Barsheshet, Adi Haber, Moshe Giladi, Uri Weinberg, Yoram Palti

**Affiliations:** Novocure Ltd, Haifa, Israel

**Keywords:** Cancer therapeutic resistance, Stress signalling

## Abstract

Tumor Treating Fields (TTFields) are electric fields that induce cancer cell death. Genomic analysis of glioblastoma tumors resected from TTFields-treated patients suggested a potential link between a reduced or absent response to TTFields and activating mutations in the phosphatidylinositol 3-kinase (PI3K) p110α subunit (PIK3CA). Our study aimed to investigate the role of the PI3K/AKT pathway in the response to TTFields. We tested changes in signaling pathways in control versus TTFields-treated U-87 MG glioblastoma, A2780 ovarian carcinoma, and H1299 non-small cell lung cancer (NSCLC) cells using the Luminex multiplex assay, validated by western blot analysis and inhibition assays. We also performed in vivo validation using immunohistochemistry on tumor sections from animals bearing orthotopic N1-S1 hepatocellular, MOSE-L ovarian, or LL/2 lung tumors that were treated with TTFields or sham. Finally, we examined the efficacy of concomitant treatment with TTFields and PI3K inhibitors in cell lines and mouse models. Our findings elucidate the mechanisms driving PI3K/AKT activation following TTFields treatment, revealing that the AKT signaling amplitude increases over time and is influenced by cell-surface and cell-cell interactions. Specifically, focal adhesion kinase (FAK) and N-cadherin were found to promote AKT phosphorylation, activating cell survival pathways. Furthermore, our investigation revealed that pharmacological inhibition of PI3K sensitized cancer cells to TTFields, both in vitro and in vivo. Our research suggests that the PI3K/AKT pathway is involved in cancer cell response to TTFields, and that inhibition of this pathway may serve as a potential therapeutic target for sensitizing cancer cells to TTFields.

## Introduction

TTFields are a cancer treatment modality based on non-invasive application of electric fields that disrupt cellular processes critical for cancer cell viability [1, 2]. Currently, TTFields therapy is approved for the treatment of patients with glioblastoma (GBM) and pleural mesothelioma, and has been shown to significantly improve median overall survival (mOS) when co-applied with standard systemic therapy in patients with metastatic non-small cell lung carcer (NSCLC) progressing on or after platinum-based therapy [3]. Ongoing research is evaluating the potential use of TTFields therapy in additional types of solid tumors [4, 5].

TTFields exert electric torque on highly polar components within cancer cells, particularly affecting spindle microtubules during mitosis [6, 7]. This interference can result in chromosome missegregation and aneuploidy, leading to cell death and slowed tumor growth [7, 8]. TTFields also alter the organization of cytoskeletal microtubules in cancer cells [9–11]. These changes activate the microtubule-associated Guanine nucleotide exchange factor H1 (GEF-H1), leading to increased levels of active Ras homolog family member A (RhoA) and Rho-associated coiled-coil kinase (ROCK) [11, 12].

A recent study by Pandey et al. suggested that activating mutations in the phosphatidylinositol 3-kinase (PI3K) p110α subunit (PIK3CA gene) are potentially associated with a diminished or absent response to TTFields, as GBM patients harboring such mutations had a significantly shorter mOS compared to patients with wild type alleles when treated with TTFields, whereas this difference was not observed in the control group [13]. PIK3CA is one of the most commonly mutated oncogenes in human cancers [14]. Hyperactivation of PI3K/AKT signaling, either through genetic alterations or activating signals, has previously been linked to tumor growth and resistance to therapy [15–17]. Inhibiting PI3K/AKT has been shown to enhance the effectiveness of chemotherapeutic agents in vitro and in vivo [18–21]. Therefore, understanding the regulation of PI3K/AKT activation in response to TTFields holds scientific and clinical importance and may reveal potential targets for concomitant treatments that can improve patient outcomes.

The main objective of this study was to identify mechanisms by which the PI3K/AKT pathway is regulated in cancer cells following TTFields treatment. We discovered two distinct mechanisms depending on cell confluence. In low-confluence cells, TTFields-induced microtubule disruption activates the GEF-H1/RhoA/ROCK axis, promoting focal adhesion formation and subsequent activation of FAK, which in turn activates the PI3K/AKT pathway. In high-confluence cells, microtubule disruption stabilize N-cadherin at adherens junctions, thereby facilitating the activation of PI3K. Based on these elucidated mechanisms, we utilized concomitant PI3K inhibition with TTFields treatment in cell cultures and animal models, demonstrating that this approach may sensitize cancer cells to TTFields treatment. Overall, these insights provide a molecular basis for understanding PI3K/AKT pathway activation in response to TTFields and offer a rationale for future clinical trials evaluating the efficacy of TTFields concurrently with PI3K inhibitors.

## Methods

### Cell culture

The following cell lines were used: A2780 ovarian carcinoma (AddexBio Technologies, C0017002); H1299 NSCLC (AddexBio Technologies, C0016053); U-87 MG GBM (AcceGen, ABC-TC1258); LL/2-Luc2 LLC (ATCC, CRL-1642-LUC2); N1-S1 HCC (ATCC, CRL-1604). MOSE-L-FFL, mouse ovarian surface epithelium cells transduced with firefly luciferase, were kindly provided by Paul C. Roberts and Eva M. Schmelz (Virginia Tech). Cells were grown in DMEM (Sartorius, 01-055-1A) or RPMI-1640 Medium (ATCC, 30-2001) media as appropriate, supplemented with 5-10% (v/v) fetal bovine serum (Thermo Fisher Scientific, 2437266), 2mM l-glutamine (Bio-West, X0550) and 50 µg/ml penicillin/streptomycin (Bio-West, L0022). Cells were routinely examined for mycoplasma contamination.

### Inhibitors

The following inhibitors were used: ROCK1/2 inhibitor Y-27632 (Abcam, ab211175); FAK inhibitor GSK2256098 (MedChemExpress, HY-100498); PI3K inhibitors BYL719 (alpleisib; Selleckchem, S2814), BKM120 (buparlisib; Selleckchem, S2247), BGT226 (Selleckchem, S2749) and GDC-0084 (paxalisib; Selleckchem, S8163); and AKT inhibitor MK-2206 (Selleckchem, S1078).

### TTFields application to cells

Cells were seeded on glass cover slips (22 mm diameter), 2 × 10^4^ cells/coverslip for 72 h experiments and 5 × 10^4^ cells/coverslip for up to 2 h experiments. After an overnight incubation, the coverslips were placed in inovitro dishes containing 2 ml media. TTFields (1.7 V/cm RMS) were applied at 200 kHz to A2780 and U-87 MG cells and at 150 kHz to H1229 cells using the inovitro^TM^ system (Fig. S1A, B) [6, 8, 22].

### Luminex assay

Lysates (500 µg) from 72 h TTFields-treated and control cells were analyzed using Luminex multiplex kits for AKT/mTOR and MAPK/SAPK pathway proteins (Merck, 48-612MAG, 48-611MAG, and 48-660MAG, respectively).

### Cell lysates and immunoblotting

Protein extracts were prepared using RIPA lysis buffer (Sigma-Aldrich; R0278) containing 150 mM sodium chloride, 1.0% IGEPAL® CA-630, 0.5% sodium deoxycholate, 0.1% SDS, and 50 mM Tris pH = 8, supplemented with a cocktail of proteases (Complete Mini, Roche; 11836153001) and phosphatase inhibitors (Thermo Scientific; 1862495). After determining the protein concentration (Bradford reagent, Bio-Rad; 23227), 30 µg of protein was resolved by SDS-polyacrylamide gel electrophoresis under reducing conditions. After electrophoresis, the proteins were transferred to polyvinylidene difluoride membranes (Bio-Rad; 1704156). Electrophoresis was performed on two different gels for each sample, one immunoblotted for total protein levels, and the other for the levels of the phosphorylated protein, using the following primary antibodies: 4E-BP1 (Cell Signaling, 9452), p4E-BP1 (Thr37/46) (Cell Signaling, 9459), AKT (Cell Signaling, 2920), pAKT (Ser473) (Cell Signaling, 4060), pAKT (Thr308) (Cell Signaling, 4056), AMPK (Cell Signaling, 2793), pAMPK (Thr172) (Cell Signaling, 2535), BAD (Abcam, ab32445), pBAD (Ser136) (Cell Signaling, 43665), FAK (Cell Signaling, 32855), pFAK (Tyr397) (Cell Signaling, 32835), GAPDH (Santa Cruz, sc-32233), GEF-H1 (Cell Signaling, 4145), pGEF-H1 (Ser886) (Cell Signaling, 14143), PI3K p85a (Cell Signaling, 13666), pPI3K p85 (Tyr458)/p55 (Tyr199) (Cell Signaling, 4228), N-cadherin (Abcam, ab18203), S6 (Cell Signaling, 4859), pS6 (S235/236) (Cell Signaling, 4858), ULK1 (Cell Signaling, 8054), pULK1 (Ser757) (Cell Signaling, 6888). Horseradish peroxidase (HRP)-conjugated secondary antibody (Abcam, ab6721 or ab97023) and a chemiluminescent substrate (Merck, WBLUF0100) were used for visualization, and signals were recorded on GeneGnome XRQ NPC gel imager (SYNGENE).

### ROCK activation assay

Cells treated with TTFields for 10 minutes were assessed using a ROCK activity commercial assay kit (Abcam, ab211175-1).

### Immunohistochemical analysis

Tumors were collected from male SD rats inoculated with N1-S1 HCC cells into the left hepatic lobe, and treated with sham-heat or 150 kHz TTFields for 5 days [23], and from sham-heat and TTFields-treated ovarian or lung tumor-bearing mice describe herein. Tumor sections were formalin-fixed, embedded, and examined immunohistochemically with an antibody against pAKT Ser473 (Cell Signaling, 3787 or 4060) and hematoxylin counterstain. Slides were scanned in an automated slide scanner 3DHistech Panoramic 259 Flash III (The Rapport Faculty of Medicine, Technion). Analysis was performed with Image J software on an average of 5 images per animal.

### ROCK/FAK inhibition assays

Cells were treated with TTFields for 2 h, with co-application of the inhibitors Y-27632 or GSK2256098 (5 µM). Treatment was terminated by cell lysis followed by western blot analysis.

### N-cadherin microscopy

Cells treated with TTFields for 72 h (or for 2 h at high cell confluency) were fixed with 4% PFA (Santa Cruz, sc-281692) for 15 min, washed with 0.1% Triton X-100 (Sigma, 9036-19-5), blocked with 10% normal donkey serum (Jackson Immunoresearch, 017-000-121) and 1% BSA (Sigma, A7906), stained overnight at 4 °C with an antibody against N-cadherin (1:1000; Abcam, ab18203), followed by 1-h incubation with Alexa Fluor 488–conjugated secondary antibody (1:200; Jackson ImmunoResearch, 711-545-152) and 0.2 µg/ml 4′,6-diamidino-2-phenylindole (DAPI) (1:500; Sigma, D9542). Images were collected using LSM 700 laser scanning confocal system, attached to an upright motorized ZeissAxio Imager Z2 microscope.

### Calcium switch assay

Cells treated with TTFields for 72 h were incubated for 30 min in media containing 4 mM EGTA (Millipore, 324626), followed by 30 min incubation in media containing 1.8 mM Ca^2+^. Cells were microscopically examined, lysed and subjected to western blot analysis.

### Immunoprecipitation assay

Lysates (400 µg proteins) of cells treated with TTFields for 72 h were precleaned by incubation with L-agarose beads (Santa Cruz, sc-2336) for 1 h at 4 °C, followed by overnight incubation at 4 °C with an antibody against N-cadherin (1 mg/ml; Abcam, ab18203). Equal amounts of non-specific IgG were added to negative control samples. Samples were incubated with A/G agarose beads (Santa Cruz, sc-2003) for 2 h at 4 °C, washed three times (Tris Base pH 7.4, 150 mM NaCl, 0.5 M EDTA, protease inhibitor, Triton-X100 and sodium vanadate), and subjected to western blot analysis.

### N-cadherin neutralization assay

Cells treated with TTFields for 72 h, serum-deprived during the last treatment day, were incubated for 1 h in the absence or presence of a monoclonal antibody against N-cadherin (1:200; Merck, C3865, clone GC4). The cells were lysed and subjected to western blot analysis.

### PI3K/AKT inhibition assays

Cells were treated with TTFields for 72 h, with co-application of alpelisib (1 nM for A2780, 250 nM for H1299). Alternatively, cells treated for 72 h were further treated with TTFields for 72 h in inhibitor-free media. Similar 144 h experiments (72 h TTFields + inhibitor, then 72 h TTFields alone) were performed in A2780 cells with buparlisib (500 nM), paxalisib (64 nM), BGT226 (8 nM), or MK-2206 (50 nM).; and in H1299 cells with MK-2206 (400 nM). In U-87 MG cells, equivalent 312 h experiments were performed (72 h TTFields + inhibitor, then 240 h TTFields alone) with paxalisib (256 nM) or BGT226 (5 nM).

Upon treatment end, cell counts were determined using the MACSQuant Analyzer flow cytometer and expressed as a percentage relative to control. For apoptosis detection, cells were stained with FITC-conjugated Annexin V (AnnV) and 7-Aminoactinomycin D (7-AAD) using a commercial kit (BioLegend, 640922), with data acquisition performed using flow cytometry. For colony formation, cells were harvested, re-plated (300 cells/well, 6-well plates), and grown for 7–14 days. Colonies were quantified after 0.5% crystal violet staining (Sigma, V5265) using ImageJ and expressed as percentages relative to control.

### In vivo assays

All animal studies were approved by the Animal Experimentation Committee according to the Ministry of Health by the Israel National Council for Animal Experimentation (approval numbers: NPC-No-IL-2110-101-4 for ovarian model; NPC-No-IL-2303-245-4 for lung model) and performed in accordance with guidelines and regulations. Animal housing and anesthesia and arrays composition and placement procedure were previously described [24]. Sample size was calculated based on a power analysis with an anticipated effect size of a 20% reduction in tumor volume. This analysis was conducted to ensure an 80% power to detect a statistically significant difference at a significance level of 0.05. Animals were stratified by initial tumor volume and then randomly assigned to treatment groups ensuring balanced group sizes and comparable average tumor sizes at baseline. Experiments were performed with 4 study groups: (1) control – sham (heat), vehicle [saline with 40% PEG300 (Selleckchem, S6704) and 5% TWEEN80 (Selleckchem, S6702)]; (2) TTFields (200 kHz ovarian model; 150 kHz lung model), vehicle; (3) sham, alpelisib (25 mg/kg/day); and (4) TTFields, alpelisib. TTFields/heat were applied to the mice torso continuously using the inovivo system (Fig. S1C, D). Alpelisib/vehicle were administered daily by oral gavage.

#### Ovarian model

Female C57Bl/6 mice, age 10–12 weeks (Envigo crs, Israel), were inoculated into the ovarian bursa with MOSE-L-FFL (5000 cells in 5 µL DMEM medium, diluted 1:1 in matrigel (Corning, CLS354263-1EA). Tumors were examined for bioluminescent (BLI) signal by In Vivo Imaging System (IVIS, Perkinelmer) 15 days after inoculation, and only mice with a total flux higher than 5 × 10^5^ (P/S) were included in the study. Animals with skin pigmentation at the area of the tumor were excluded. Upon treatment end the tumors were re-examined for their BLI signal, and tumor volume fold increase was calculated.

#### Lung model

Male C57Bl/6 mice, age 10–12 weeks (Envigo crs, Israel), were inoculated into the lungs with LL/2 Lewis lung carcinoma cells (4500 cells in 5 µL RPMI medium, diluted 1:1 in matrigel). Tumors were examined by magnetic resonance imaging (MRI, Bruker) 6 days after inoculation, and only mice with verified localized tumors were included in the study. Upon treatment end the tumors were weighed and measured with Vernier calipers using the formula width^2^ × length × 0.5.

### Statistical analysis

Data are presented as mean ± standard error of the mean (SEM) for in vitro and mean ± standard deviation (SD) for in vivo. Data normality was evaluated using Shapiro–Wilk test. Statistical significance (*p < 0.05, **p < 0.01, and ***p < 0.001) was calculated using GraphPad Prism 10 software (La Jolla). Statistical tests are specified along with figure panels and used according to the type of analysis.

## Results

### TTFields application activates the PI3K/AKT signaling pathway in cancer cells

**Fig. 1:**
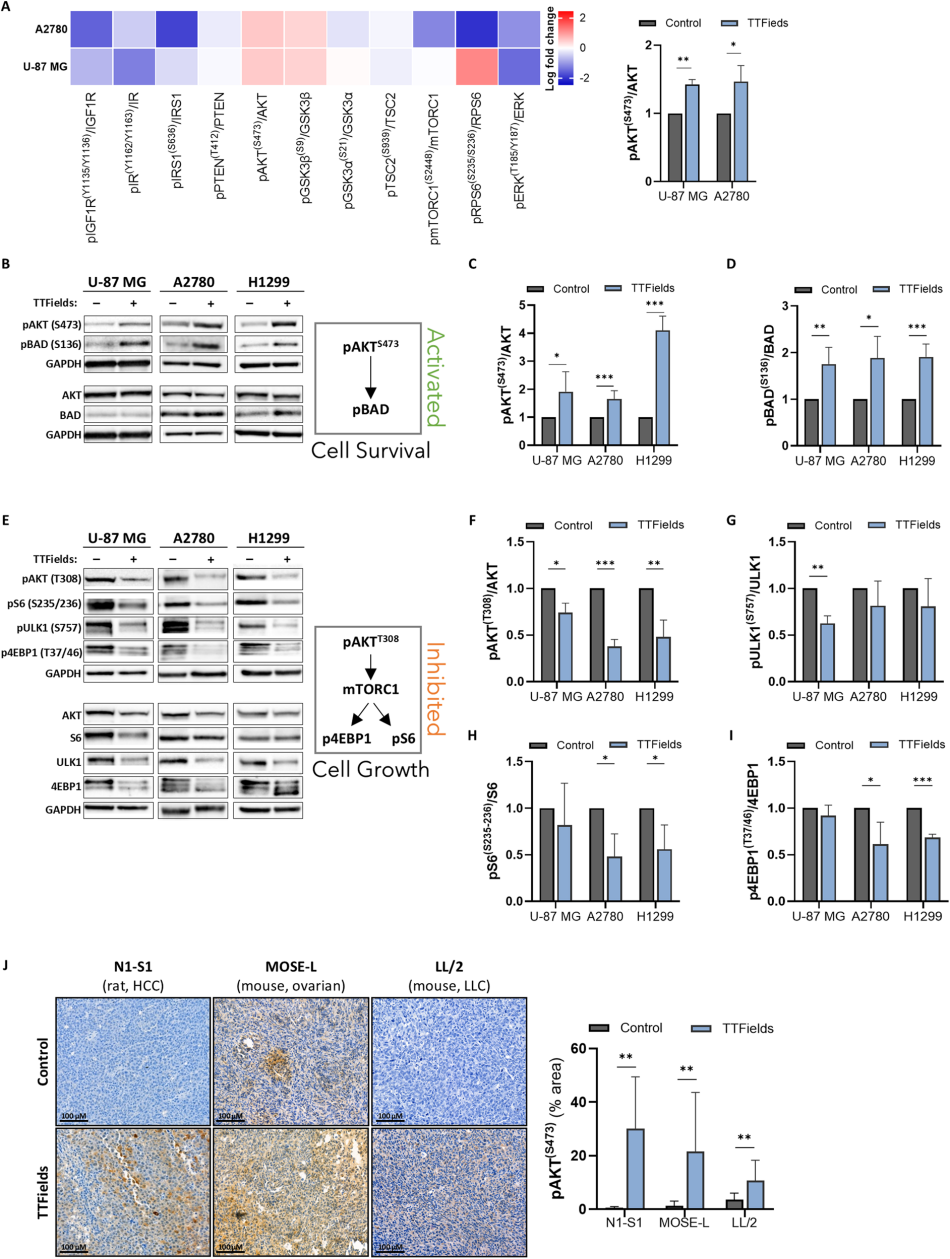
TTFields application activates the PI3K/AKT signaling pathway in cancer cells. **A** Luminex assay of lysates from control and TTFields-treated (72 h) U-87 MG and A2780 cells. Left panel: heatmap showing the log fold change of phosphorylated to total protein ratio relative to the control. Right panel: AKT phosphorylation (Ser473) is presented as mean ± SEM; *N* ≥ *2*. *p < 0.05; **p < 0.01; multiple unpaired t-tests. **B**–**D** Western blot analysis of AKT, pAKT (Ser473), BAD, pBAD (Ser136), and GAPDH in lysates from control and TTFields-treated (72 h) U-87 MG, A2780, and H1299 cells. Densitometric analysis for phosphorylation fold change is shown as mean ± SEM; *N* ≥ 3. *p < 0.05, **p < 0.01, and ***p < 0.001; multiple unpaired t-tests. **E**–**I** Western blot analysis of AKT, pAKT (Thr308), ULK1, pULK1 (Ser757), S6, pS6 (Ser235/236), 4EBP1, 4EBP1 (Thr37/46), and GAPDH in lysates from control and TTFields-treated (72 h) U-87 MG, A2780, and H1299 cells. Densitometric analysis for phosphorylation fold change is shown as mean ± SEM; *N* ≥ 3. *p < 0.05, **p < 0.01, and ***p < 0.001; multiple unpaired *t*-tests. **J** Immunohistochemical analysis of pAKT (Ser473) in tumor sections from control and TTFields-treated animals: N1-S1 hepatocellular carcinoma (HCC) tumors (150 kHz, 5 days), MOSE-L ovarian cancer tumors (200 kHz, 10 days), and LL/2 lung tumors (150 kHz, 8 days). Representative images and quantification performed as an average of 5 images per animal, shown as mean ± SD with a total of 7–13 animals per group. **p < 0.01; multiple unpaired *t*-tests.

TTFields have been shown to induce cancer cell death in vitro and to inhibit tumor growth in vivo in a variety of tumor models [8, 23, 25–30]. To investigate the possible involvement of the PI3K/AKT pathway in the response to TTFields, we assessed signaling alterations in cells exposed to TTFields for 72 h using Luminex multiplex analysis (Figs. 1A and S2). TTFields treatment resulted in increased AKT phosphorylation at Ser473 without affecting the phosphorylation levels of extracellular signal-regulated kinase 1 and 2 (ERK1/2). These findings suggest that PI3K/AKT signaling predominates over RAF/MEK/ERK signaling in mediating cellular responses to TTFields. The Luminex multiplex analysis also indicated no activation of insulin-like growth factor 1 receptor (IGF1R) and insulin receptor (IR) upstream of AKT.

Western blot analysis confirmed an increase in phosphorylation of AKT on Ser473 in U-87 glioblastoma cells, A2780 ovarian cancer cells, and H1299 lung cancer cells (Fig. 1B, C). In correlation, phosphorylation levels of the p85 regulatory subunit of PI3K (Tyr458) were also increased (Fig. S3**)**. Of note, the lowest response was observed in the A2780 cells, which harbors a PIK3CA activating mutation [31]. While TTFields promoted phosphorylation of AKT on Ser473, phosphorylation at Thr308 was inhibited in all the tested cell lines (Fig. 1E, F). Taken together, these findings indicate that TTFields differentially regulate Ser473 and Thr308 phosphorylation and that the ratio between the two residues could be important in determining downstream substrate specificity.

Next, we tested survival signals downstream of AKT. We observed increased phosphorylation of BCL2-associated agonist of cell death (BAD; Ser136) (Fig. 1B, D), which promotes cell survival when phosphorylated [32]. The Luminex multiplex analysis **(**Figs. 1A and S2) indicated no activation of mammalian target of rapamycin complex 1 (mTORC1) downstream of AKT. To clarify the role of mTORC1 in cell survival under TTFields application, we assessed the phosphorylation status of several mTORC1 substrates, including Unc-51-like autophagy-activating kinase 1 (ULK1; Ser757), ribosomal protein S6 (S6; Ser235/236), and 4E binding protein 1 (4EBP1; Thr37/46), and observed decreased phosphorylation of all three proteins (Fig. 1E, G–I). AMP-activated protein kinase (AMPK) affects different targets to suppress mTORC1 [33]. Prior work on glioma cells (including U-87 MG) has revealed that the application of TTFields resulted in AMPK pathway activation [34]. Therefore, we examined AMPK phosphorylation (Thr172) in A2780 ovarian and H1299 lung cancer cells and found that it was increased (Fig. S4). These results suggest that the mTORC1 pathway is negatively regulated by AMPK activation under TTFields.

Next, we performed immunohistochemical analysis of tumor sections collected from animal models, in which TTFields were previously shown to inhibit tumor growth [23, 28, 29]. Increased AKT phosphorylation following TTFields application was observed in all examined models: N1-S1 hepatocellular carcinoma (HCC), MOSE-L ovarian cancer, and LL/2 lung tumors (Fig. 1E). This confirmed that TTFields-induced AKT activation observed in cell cultures is relevant in vivo.

### Focal adhesion kinase (FAK) functions upstream of AKT activation following TTFields application

**Fig. 2:**
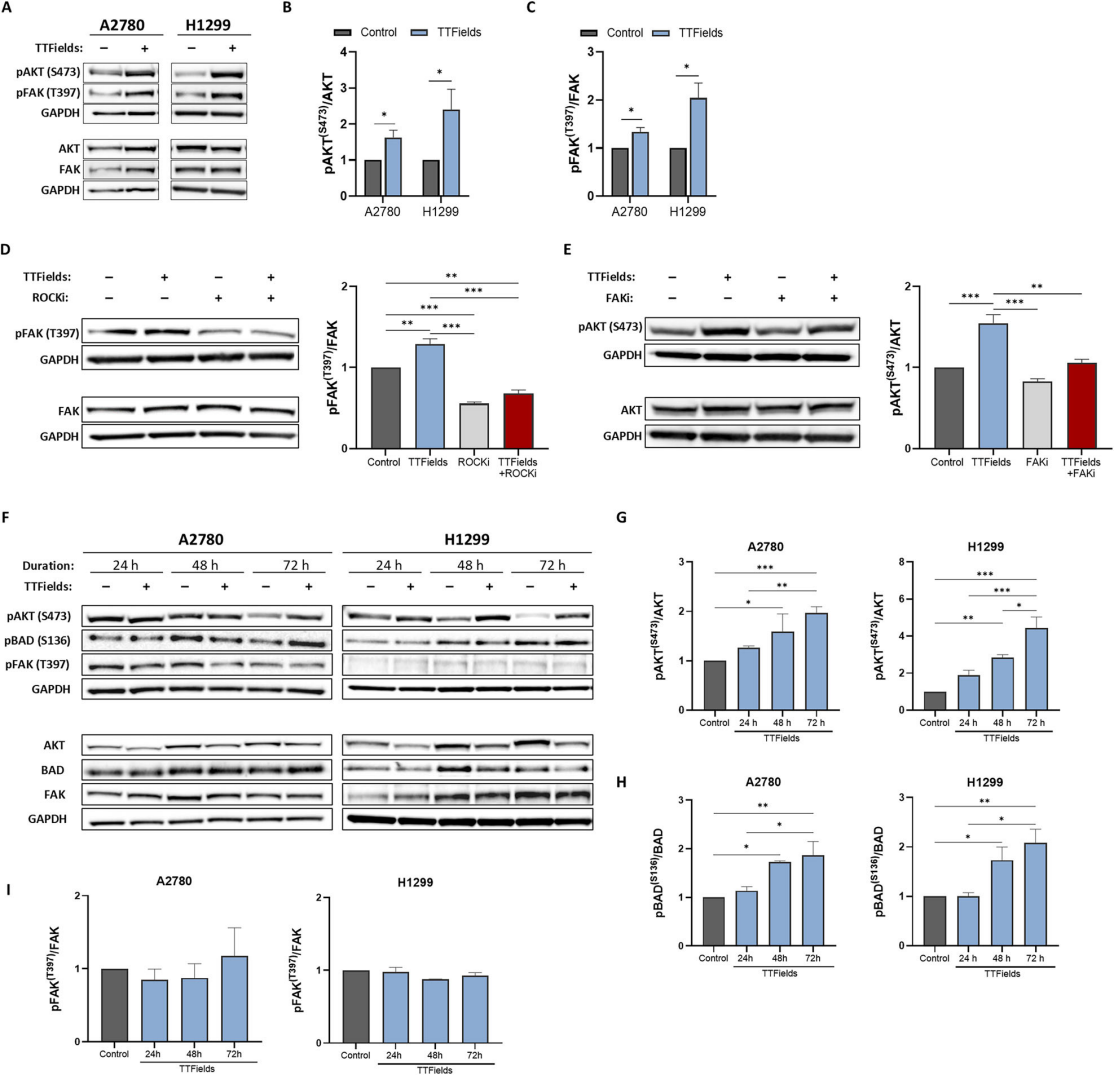
FAK functions upstream of AKT activation following TTFields application. **A**–**C** Western blot analysis of AKT, pAKT (Ser473), FAK, pFAK (Tyr397), and GAPDH in lysates from control and TTFields-treated (2 h) A2780 and H1299 cells (**A**). Densitometric analysis for phosphorylation fold change is shown as mean ± SEM; *N* ≥ 3. *p < 0.05, **p < 0.01, and ***p < 0.001; multiple unpaired t-tests. Western blot analysis of FAK, pFAK (Tyr397), AKT, pAKT (Ser473), and GAPDH in lysates from control and TTFields-treated (2 h) H1299 cells in the presence of ROCK inhibitor (ROCK_i_) (**D**) or FAK inhibitor (FAK_i_) (**E**). Densitometric analysis for phosphorylation fold change is shown as the mean ± SEM; *N* ≥ *3*. *p < 0.05, ** *p* < 0.01, and ***p < 0.001; one-way ANOVA followed by Tukey’s post hoc test. **F**-**I** Western blot analysis of AKT, pAKT (Ser473), BAD, pBAD (Ser136), FAK, pFAK (Tyr397), and GAPDH in lysates from control and TTFields-treated (24, 48, and 72 h) A2780 and H1299 cells. Densitometric analysis for phosphorylation fold change is shown as mean ± SEM; *N* ≥ 3. *p < 0.05, **p < 0.01, and ***p < 0.001; one-way ANOVA followed by Tukey’s post hoc test.

To elucidate the signaling cascade leading to AKT activation in response to TTFields treatment, we performed western blot analysis following short-term TTFields application. This revealed that AKT was phosphorylated at Ser473 shortly after the start of TTFields application **(**Figs. 2 and S5A, B). Previous studies demonstrated that TTFields can activate the GEF-H1/RhoA/ROCK signaling pathway within minutes of application [11, 12], resulting in larger and more numerous focal adhesions [11]. Focal adhesions provide a scaffold for integrating growth factor signaling and integrin pathways [35]. Once recruited to focal adhesions, FAK becomes catalytically active [36], and may subsequently activate the PI3K/AKT signaling pathway [37, 38]. Therefore, we tested the activation of FAK and the GEF-H1/RhoA/ROCK signaling pathway in TTFields-treated cells. We found increased FAK phosphorylation (Tyr397) **(**Figs. 2A, C and S5A, C), ROCK activation (Fig. S5D), and GEF-H1 phosphorylation (Ser886) (Fig. S5E), which were evident as early as 10 min after the start of TTFields treatment. Inhibition of ROCK significantly decreased FAK phosphorylation relative to TTFields treatment alone and inhibition of FAK abrogated TTFields-induced AKT (Ser473) phosphorylation (Fig. 2D, E), validating the involvement of the GEF-H1/RhoA/ROCK pathway in cellular response to TTFields.

To elucidate the kinetics of AKT activation during TTFields treatment, we evaluated changes in AKT phosphorylation after 24, 48, and 72 h. We observed an incremental increase in AKT (Ser473) phosphorylation over time (Fig. 2F, G), accompanied by a time-dependent increase in BAD phosphorylation (Fig 2F, H). Notably, while FAK phosphorylation increased after 2 h of TTFields treatment (Fig. 2A, C), its levels remained similar to those of the control cells after longer treatment durations (Fig. 2F, I). To examine the influence of cell confluency on cellular response to TTFields, we examined AKT activation via the FAK axis following 2 h TTFields treatment of cells at high cell confluency (at a confluency equivalent to that attained at the end of the 72 h experiments). Our observations suggest that AKT (Ser473) was phosphorylated under these conditions, whereas FAK was not (Fig. S6A–C).

Overall, these results suggest that, while FAK activation mediates immediate response to TTFields in a GEF-H1/RhoA/ROCK-dependent manner when cells are in low confluency, this axis is not involved in the response to TTFields at high cell confluency.

### N-cadherin engagement functions upstream of AKT activation during TTFields application

**Fig. 3:**
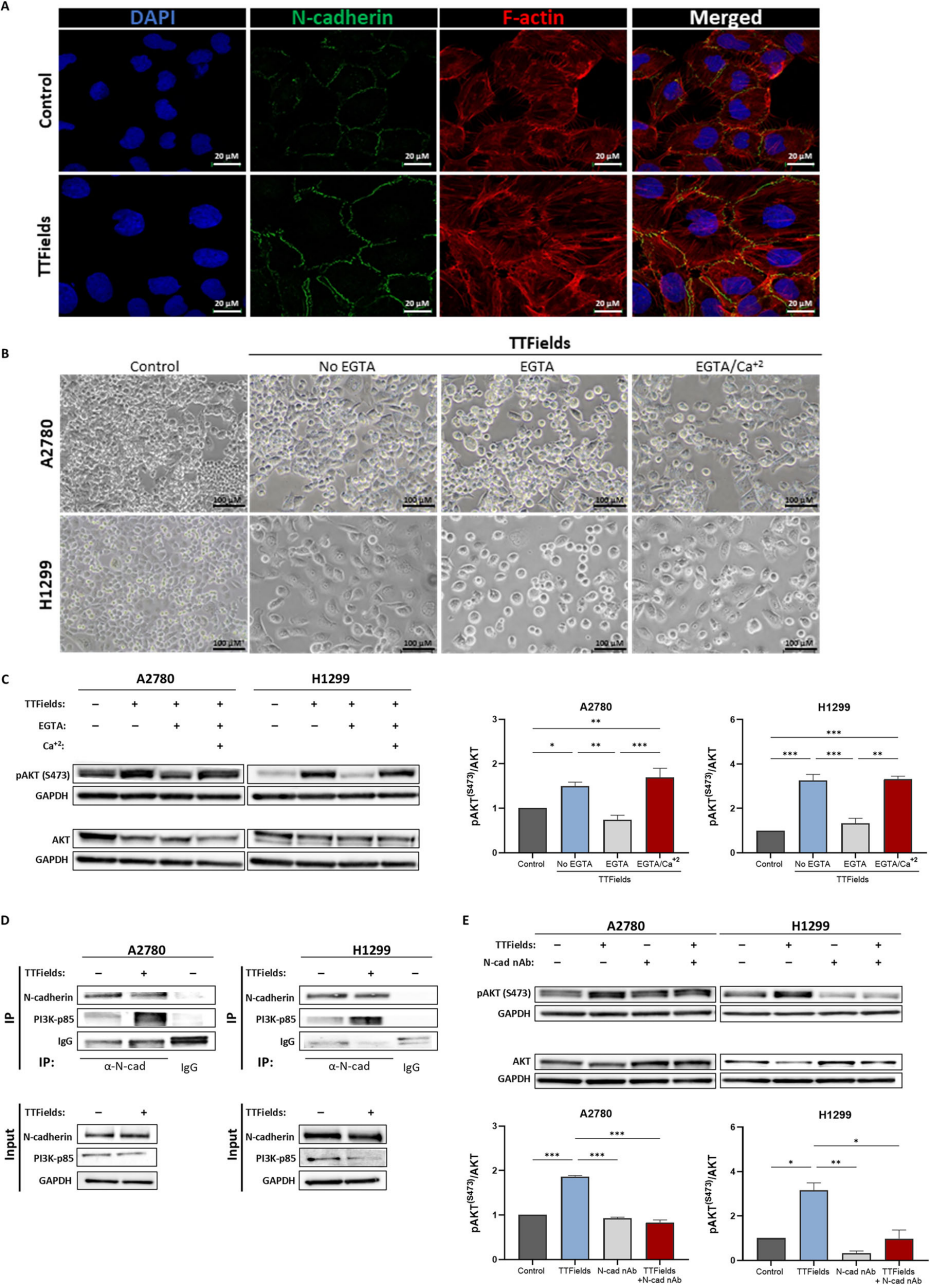
N-cadherin engagement functions upstream of AKT activation during TTFields application. **A** Confocal fluorescence microscopy images (×40 magnification) of N-cadherin in control and TTFields-treated (72 h) H1299 cells. **B**, **C** Representative phase contrast images and western blot analysis of AKT, pAKT (Ser473), and GAPDH in lysates from control and TTFields-treated (72 h) A2780 and H1299 cells, subsequently not treated (No EGTA), treated with EGTA for calcium elimination (EGTA), or treated with EGTA followed by serum-free calcium-containing medium for calcium reintroduction (EGTA/Ca^+2^). Densitometric analysis for phosphorylation fold change is shown as the mean ± SEM; *N* ≥ *3*. * *p* < 0.05, and ***p < 0.001; one-way ANOVA followed by Tukey’s post hoc test. **D** Western blot analysis of N-cadherin and PI3K p85 subunit in cell lysates (input) and immunoprecipitants (IP) pulled down with an antibody against N-cadherin (α-N-cad) from control and TTFields-treated (72 h) A2780 and H1299 cells. **E** Western blot analysis of AKT, pAKT (Ser473), and GAPDH in lysates from control and TTFields-treated (72 h) A2780 and H1299 cells, with or without an N-cadherin neutralizing antibody (N-cad nAb). Densitometric analysis for phosphorylation fold change is shown as the mean ± SEM; *N* = *2*. *p < 0.05, **p < 0.01, and ***p < 0.001; one-way ANOVA followed by Tukey’s post hoc test.

The observation that AKT (Ser473) phosphorylation following TTFields application occurs in the absence of FAK activation at high cell confluency prompted us to explore the involvement of cadherins, implicated in the activation of the PI3K/AKT pathway through cell-cell contacts [39–43]. Confocal microscopy following 72 h TTFields application revealed colocalization of N-cadherin and F-actin at cell-cell interfaces, with increased levels of N-cadherin on the membrane of treated cells (Fig. 3A). Elevation of N-cadherin on the cell membrane was also evident following 2 h TTFields application to cells at high confluency (Fig. S6D). Cells require Ca^+2^ to establish cadherin homophilic ligation [40, 44]; therefore, we next eliminated Ca^+2^ ions from the culture media by addition of a chelating agent (EGTA), followed by reintroduction of Ca^+2^ ions for restoration of cell-cell contacts (Fig. 3B, C). Calcium elimination from TTFields-treated cells resulted in the adoption of a rounded morphology and reduced phosphorylation of AKT (Ser473) compared to TTFields-treated cells under normal calcium levels. Re-engagement of cell-cell adhesion by calcium restoration resulted in a slight reversal of the rounded phenotype and reinstated AKT (Ser473) phosphorylation to a level equivalent to TTFields without Ca^+2^ depletion (TTFields/No EGTA group).

Next, we tested the recruitment of the p85 regulatory subunit of PI3K to the N-cadherin complexes by pull-down assays using an antibody against N-cadherin. As we did not distinguish between membrane and cytosolic N-cadherin, we found comparable levels of N-cadherin in the control and TTFields-treated cells (Fig. 3D). However, the PI3K-p85 subunit was found to be associated with N-cadherin following TTFields treatment, suggesting an increased recruitment of PI3K to N-cadherin complexes. The negative control (non-specific antibody) showed lack of precipitation of N-cadherin or PI3K-p85. We then utilized an N-cadherin neutralizing antibody to eliminate N-cadherin homophilic ligation and inhibit N-cadherin-mediated cell-cell contact. The addition of the neutralizing antibody to TTFields treated cells resulted in a significant reduction in AKT (Ser473) phosphorylation compared to the absence of antibody (Fig. 3E).

Overall, these findings suggest that N-cadherin-mediated cell-cell contacts initiate PI3K-dependent signal transduction, leading to FAK-independent activation of AKT (Ser473) during prolonged TTFields exposure.

### Inhibition of PI3K/AKT activation enhances the efficacy of TTFields treatment

**Fig. 4:**
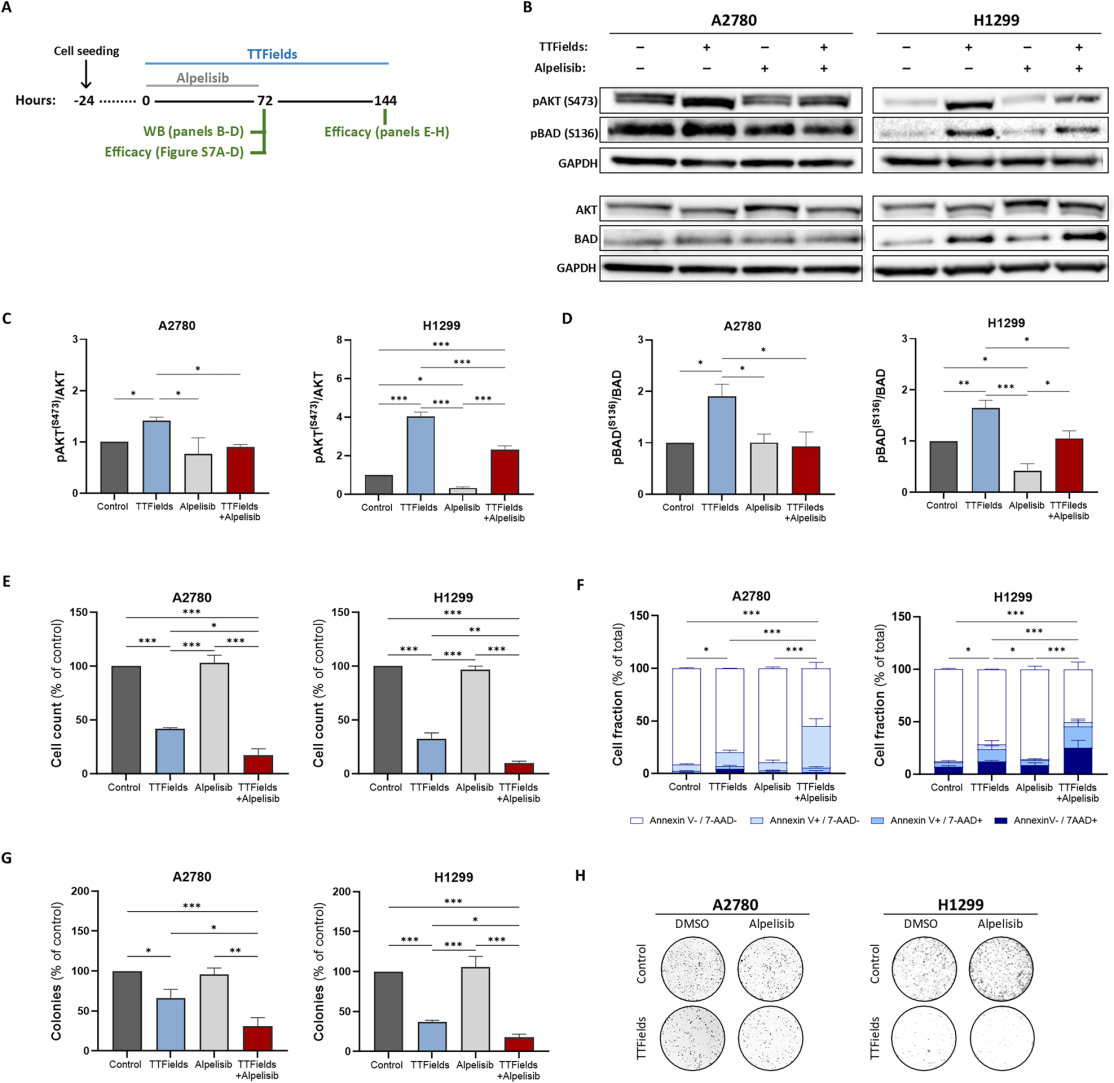
Inhibition of PI3K/AKT activation enhances the in vitro efficacy of TTFields treatment. **A** Experimental timeline for treatment with TTFields and alpelisib. **B**–**D** Western blot analysis of AKT, pAKT (Ser473), BAD, pBAD (Ser136), and GAPDH in lysates from control and TTFields-treated (72 h) A2780 and H1299 cells, with or without concomitant alpelisib (72 h). Densitometric analysis for phosphorylation fold change is shown as mean ± SEM; *N* ≥ 3. *p < 0.05, **p < 0.01, and ***p < 0.001; one-way ANOVA followed by Tukey’s post hoc test. **E**–**H** Cell counts, apoptosis [Live: Annexin V−/7AAD−; early apoptosis: Annexin V+/7AAD-; late apoptosis: Annexin V+/7AAD+], and colony formation of control and TTFields-treated (144 h) A2780 and H1299 cells, with or without concomitant alpelisib (during the first 72 h). Results shown are as mean ± SEM; *N* ≥ *3*. * < 0.05, **p < 0.01, and ***p < 0.001; one-way ANOVA followed by Tukey’s post hoc test for cell count and clonogenic survival; two-way ANOVA followed by Tukey’s post hoc test for apoptosis, with significance within shown for the live cell fraction.

The finding that PI3K/AKT signaling is involved in the response of cancer cells to TTFields prompted us to explore the effects of pharmacological inhibition of this pathway on the responsiveness of cancer cells to TTFields cytotoxicity (Fig. 4A). For this purpose, we used the α-isoform-specific PI3K inhibitor, alpelisib, at IC_25_ (Fig. S7).

**Fig. 5:**
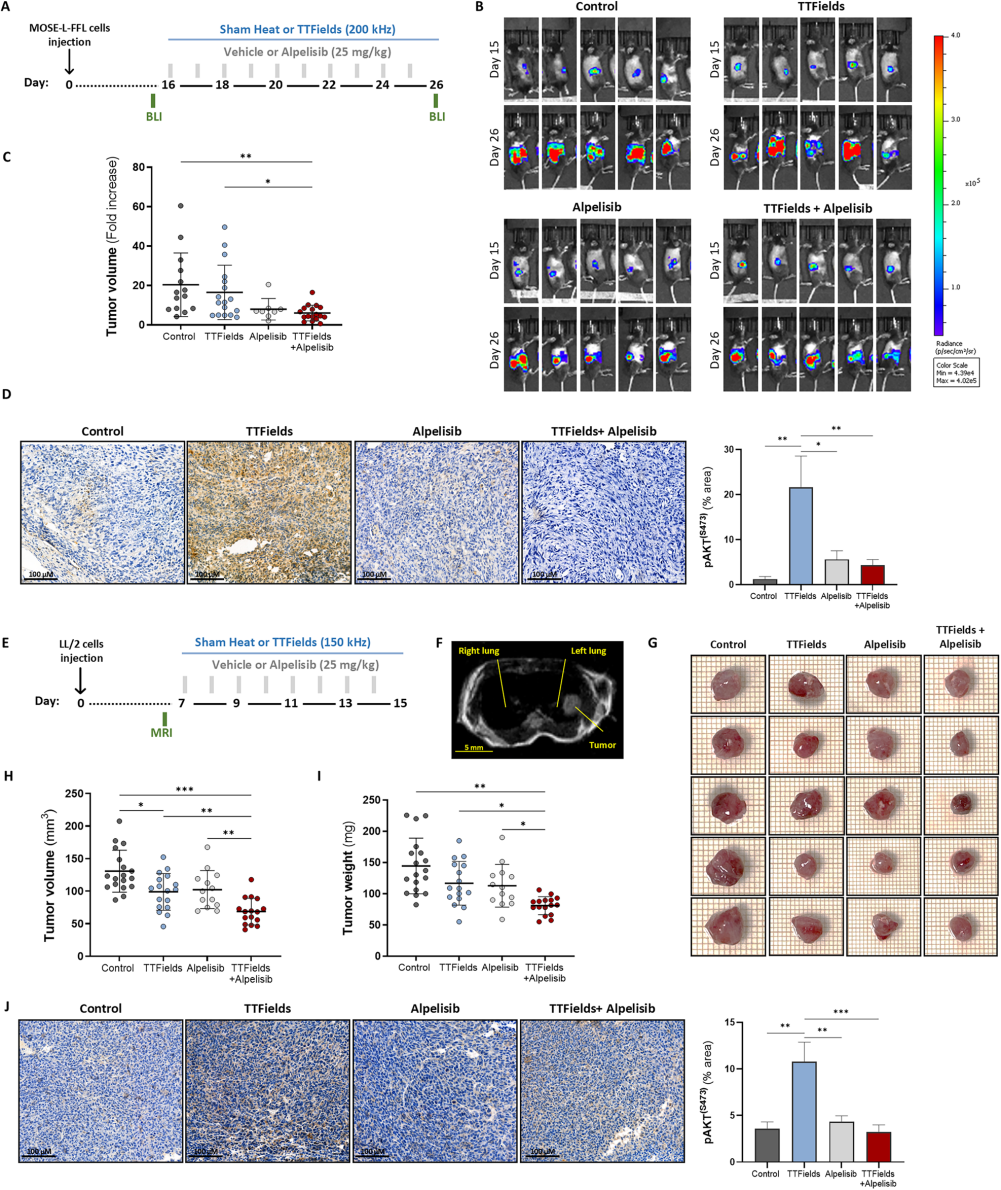
Inhibition of PI3K/AKT activation enhances the in vivo efficacy of TTFields treatment. **A** Experimental timeline for treatment of ovarian tumor-bearing mice with TTFields and alpelisib (3 independent experiments, total of 8-17 mice/group). **B** Representative bioluminescent images. **C** Tumor volume fold change calculated from bioluminescent signals. **D** Immunohistochemical analysis of pAKT (Ser473) in tumor sections representative images, quantification performed as an average of 5 images per animal, and shown as the mean ± SD; *p < 0.05, and **p < 0.01; one-way ANOVA followed by Tukey’s post hoc test. The data for the control and TTFields treatment groups are shown also in Fig. 1J. **E** Experimental timeline for the treatment of lung tumor-bearing mice with TTFields and alpelisib (3 independent experiments, total of 13–18 mice/group). **F** Representative MRI image. **G** Representative tumor images. **H** Tumor volumes, measured using Vernier calipers. **I** Tumor weights. **J** Immunohistochemical analysis of pAKT (Ser473) in tumor sections representative images, quantification performed as an average of 5 images per animal, and shown as mean ± SD; *p < 0.05, **p < 0.01, and ***p < 0.001; one-way ANOVA followed by Tukey’s post hoc test. The data for control and TTFields treatment groups are shown also in Fig. 1J.

First, we confirmed that alpelisib inhibited TTFields-induced phosphorylation of AKT (Ser473) (Fig. 4B, C) and BAD (Fig. 4B, D). While this experimental setup, with 72 h treatment duration, did not reduce cell counts, it resulted in increased apoptosis and reduced clonogenic potential compared to TTFields monotherapy, although in most cases, it did not reach statistical significance (Fig. S8). These results encouraged us to explore whether extending TTFields treatment duration may reduce cell counts, as suggested by the observation of reduced clonogenic potential. The following setup was used (Fig. 4A): TTFields concurrent with alpelisib for 72 h, followed by drug washout and continued TTFields treatment for an additional 72 h. Here, co-treatment with TTFields and alpelisib resulted in a significant reduction in cell counts compared to all other groups, accompanied by increased apoptosis and reduced clonogenic potential for co-application of TTFields and alpelisib compared with TTFields monotherapy (Fig. 4E–H). Similar responses were also evident when utilizing the pan-selective PI3K inhibitor buparlisib and the dual PI3K/mTOR inhibitors BGT226 and paxalisib (however, the benefit was milder with paxalisib), as well as with the AKT inhibitor MK-2206 (Fig. S9). Overall, these results suggest that PI3K/AKT pathway inhibition may enhance the anti-cancer activity of TTFields. We further examined the effects of concomitant TTFields and alpelisib treatment in two orthotopic tumor models. For ovarian tumor-bearing mice, the tumor fold change was calculated by bioluminescent signal measurements at the start and end of treatment (Fig. 5A–C). While TTFields alone demonstrated only a minor decrease in tumor volume, alpelisib alone exhibited a stronger effect, but it was not significant relative to control mice. However, a significant reduction in tumor growth was observed in the mice treated with TTFields and alpelisib relative to the control mice and to mice treated with TTFields monotherapy. Treatment with TTFields significantly increased AKT (Ser473) phosphorylation levels in tumor sections, whereas co-treatment with alpelisib completely eliminated this effect (Fig. 5D).

In lung tumor-bearing mice, tumor localization in the lung was verified at the start of treatment using MRI, and tumor volume and weight were examined at the end of treatment (Fig. 5E, F). Unlike the ovarian cancer model, TTFields alone significantly reduced tumor growth, with a relatively similar magnitude of effect to that of alpelisib alone. The co-application of TTFields and alpelisib had enhanced efficacy, with tumor volume and weight being significantly lower than those in all other treatment groups (Fig. 5G–I). Similar to the ovarian cancer model, TTFields significantly increased AKT (Ser473) phosphorylation levels in tumor sections, and co-treatment with alpelisib abolished this effect (Fig. 5J).

Collectively, our in vitro and in vivo studies suggest that cancer cells may be sensitized to TTFields by the co-administration of the PI3K inhibitor alpelisib.

## Discussion

Reduced sensitivity to treatment arising from constitutive or inducible AKT activity has been extensively studied following the application of different anticancer modalities, including chemotherapy, radiotherapy, targeted therapy, and immunotherapy [18, 45, 46]. Combination with inhibitors of this pathway results in sensitization and enhanced treatment efficacy [18–21]. Similarly, in a recent study, the response to TTFields was shown to be compromised by activating mutations in PIK3CA [13]. In the current study, we found increased AKT phosphorylation following application of TTFields, in vitro and in vivo, indicating that inducible activation of the PI3K/AKT pathway may also affect the response to TTFields treatment.

How does the PI3K/AKT axis ultimately contribute to the activation of survival signals under TTFields? One of the most common downstream effectors of AKT is mTORC1, which integrates several proteins to promote cancer cell survival [47]. Our study reveals that TTFields selectively induce phosphorylation of AKT at Ser473, without concomitant activation of the mTORC1 pathway. This observation is noteworthy because phosphorylation of AKT at Thr308 is crucial for mTORC1 signaling, whereas Ser473 phosphorylation primarily governs anti-apoptotic and cell survival pathways [48, 49]. We observed no evidence of mTORC1 activation, as assessed by examining downstream targets that serve as reliable indicators of mTORC1 activity. Notably, AMPK, a known inhibitor of mTORC1 [33, 50], was activated during TTFields exposure. This finding, coupled with our previous observation of TTFields induce AMPK-dependent autophagy [34], further supports the absence of mTORC1 activation. Moreover, we found that TTFields promote phosphorylation of BAD at Ser136, a modification associated with cell survival [51]. These findings suggest that TTFields promote cell survival through mTOR-independent mechanisms. Future studies should examine other major effectors downstream of the PI3K/AKT signaling pathway (e.g. Forkhead box Os (FOXOs) or MDM2) that may also be involved in regulating the response to TTFields. Future studies focused on the validation of these potential downstream pathways will likely further elucidate the TTFields response mechanisms.

What pathways are involved in the activation of AKT (Ser473) in response to TTFields? Microtubules have previously been shown to be affected by TTFields [11, 12]. Our results suggest that TTFields-induced microtubule disruption leads to the activation of the GEF-H1/RhoA/ROCK signaling pathway, resulting in the activation of AKT (Ser473) downstream of FAK. Although FAK plays a major role in response to TTFields application at low cell confluency, we found that FAK was not involved in the augmentation of AKT (Ser473) signal amplitude over time, as cell density increased. Further studies are needed to explore other mechanisms by which microtubules might act as upstream regulators of the PI3K signaling cascade, for example, through the spatial regulation of antagonists, such as PTEN, or by direct participation in receptor substrate recruitment [52–54].

Our study revealed the involvement of the N-cadherin complex in AKT (Ser473) activation in response to TTFields treatment at high cell confluency. N-cadherin is typically expressed in nervous system cells and is scarce in normal cells. However, it has recently been shown to be aberrantly expressed in cancer cells and is correlated with a poor prognosis [55]. The high levels of N-cadherin in tumors may potentially circumvent TTFields in vivo efficacy, highlighting the importance of examining the possible co-application of TTFields with agents that inhibit the PI3K/AKT pathway.

Concomitant treatment with TTFields and the PI3K inhibitor alpelisib reduced AKT (Ser473) phosphorylation and enhanced TTFields efficacy across tumor types, as demonstrated in vitro for GBM, ovarian cancer, and lung cancer models, and in vivo for ovarian and lung cancer models. Unfortunately, in vivo testing in a GBM model was not possible because of the lack of TTFields arrays for treating the mouse head, and the fact that alpelisib does not cross the blood-brain barrier. The magnitude of effect of TTFields was different in the different tumor models. Nevertheless, the efficacy of TTFields was enhanced by the co-administration of the inhibitor. These results indicate that the activation of AKT (Ser473) plays a pivotal role in limiting the antitumor activity of TTFields and that PI3K inhibition has the potential to enhance the therapeutic effects of TTFields. This co-application approach may be particularly relevant for patients with activating mutations in PIK3CA (~30% in a variety of cancers) as it could overcome the resistance associated with these mutations [13, 56]. Additionally, PIK3CA is one of the most commonly mutated oncogenes in human cancers [14], and PIK3CA mutations have been shown to be a favorable factor for the clinical outcomes of PI3K inhibitors [57].

A previous study by Elkabets et al. demonstrated that inhibition of mTORC1 is needed for optimal antitumor activity by PI3K inhibitors, such as alpelisib, that signaling through the mTORC1 pathway is associated with resistance to PI3K inhibitors, and that simultaneous administration of mTORC1 and PI3K inhibitors may enhance the efficacy of the latter and delay resistance development [58]. As our data suggest that TTFields application activates AKT (Ser473) without downstream activation of mTORC1, it would be of interest to explore whether TTFields may circumvent mTORC1 function as a bottleneck for the efficacy of PI3K inhibitors, or whether concomitant treatment with dual PI3K and mTOR inhibition may still be required.

**Fig. 6:**
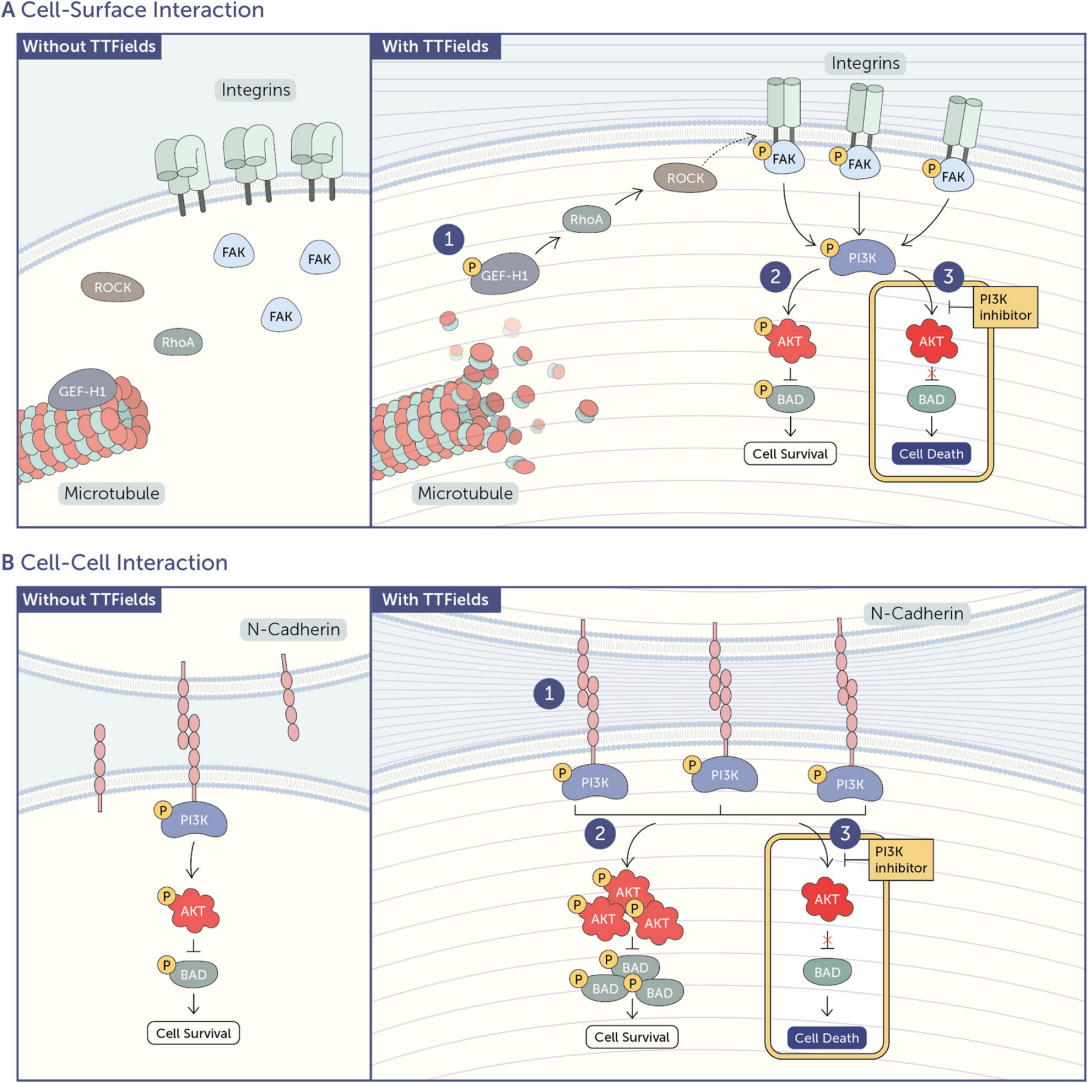
Schematic representation of the key mechanisms involved in TTFields-induced activation of AKT. **A** Activation through cell-surface interactions. Without treatment, the catalytic activity of GEF-H1 is attenuated by microtubule binding. TTFields exert directional forces on microtubules, leading to their disassembly and hence to the release of GEF-H1, promoting activation of the GEF-H1/RhoA/ROCK signaling pathway, leading to the formation of focal adhesions and activation of the downstream target FAK (1). This, in turn, leads to activation of the PI3K/AKT axis, with downstream inactivation of the pro-apoptotic protein BAD and subsequent cell survival (2). Co-application of TTFields with an inhibitor of the PI3K/AKT pathway blocks cell survival signals, sensitizing tumor cells to TTFields-induced cell death (3). **B** Activation through cell-cell interactions. Without treatment, N-cadherin forms adherens junctions between adjacent cells and triggers PI3K/AKT signaling. Exposure to TTFields potentiates multiple homophilic ligations of N-cadherin, thus elevating intercellular N-cadherin interactions (1), increasing PI3K/AKT signal amplitude, and augmenting BAD inactivation, consequently leading to cell survival (2). Co-application of TTFields with an inhibitor of the PI3K/AKT pathway blocks the cell survival signals, sensitizing the tumor cells to TTFields-induced cell death (3). Protein phosphorylation sites: GEF-H1, Ser886; FAK, Tyr397; PI3K p85, Tyr458; AKT, Ser473; BAD, Ser136.

Overall, this study offers valuable insights into the molecular mechanisms governing the activation of the PI3K/AKT signaling pathway in response to TTFields. We found that the response varies depending on cell confluency: FAK activation by means of microtubule disruption and activation of the GEF-H1/RhoA/ROCK axis at low confluency and N-cadherin stabilization at adherens junctions at high confluency. These findings underscore the critical roles of cell-surface and cell-cell interactions in this process, as illustrated in Fig. 6. Furthermore, our study presents a potential therapeutic strategy to sensitize cancer cells to TTFields treatment via co-application with PI3K inhibitors. Overall, these findings provide a molecular basis for understanding PI3K/AKT pathway activation in response to TTFields, and hold promise for improving the outcomes of TTFields therapy.

## Supplementary information

Supplementary Material

## Data Availability

All data generated or analyzed during this study are included in this published article and its supplementary file. Further inquiries can be directed to the corresponding authors.
