# Supplementary material for: Role of the PI3K/AKT signaling pathway in the cellular response to Tumor Treating Fields (TTFields)
Source: Cell Death Dis. 2025 Mar 27;16(1):210. doi: 10.1038/s41419-025-07546-8 (PMC11950169; doi:10.1038/s41419-025-07546-8)
Supplement: Supplementary file 1 — Supplementary Material [file 41419_2025_7546_MOESM1_ESM.pdf]

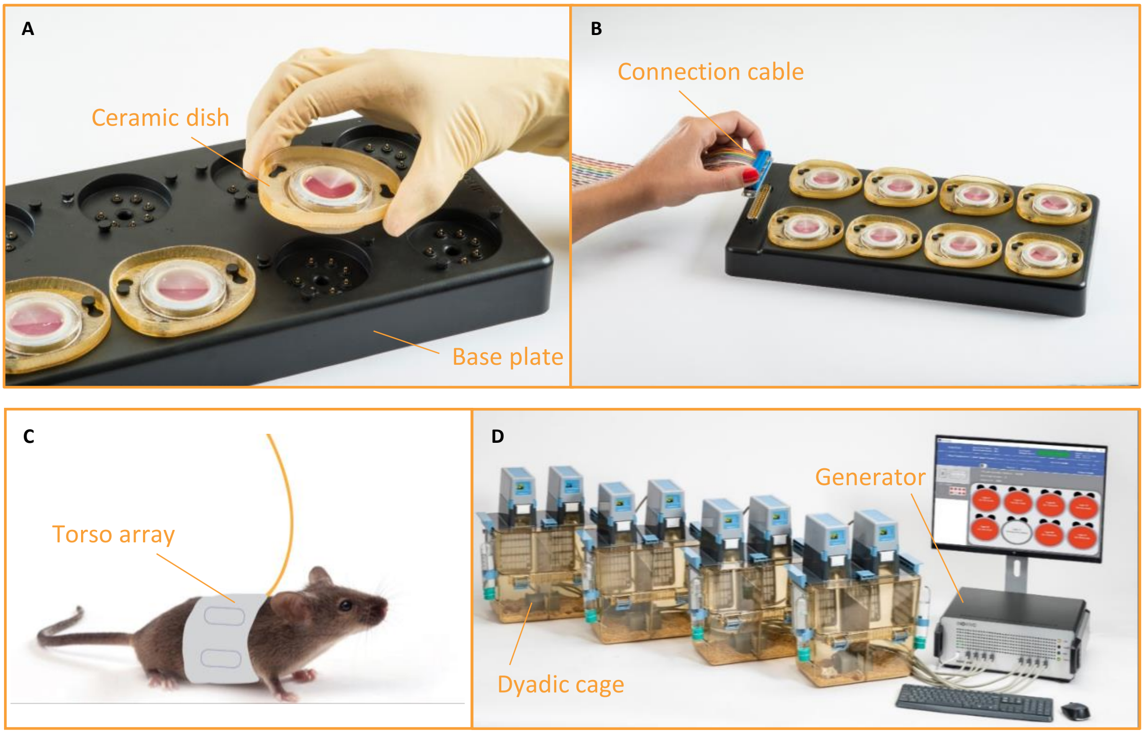

Figure S1. Lab tools for applying TTFields to cells and mice.
(A-B) The inovitro system for applying TTFields to cells. Cells are seeded in individual ceramic dishes that are connected to the base plate, which is connected via a cable to the electric field generator (not shown). **(C-D)** The inovivo system for applying TTFields to mouse torso. Mice are wrapped around their torsos with electrodes and placed within dedicated cages allowing individual housing while maintaining dyadic interaction. The electrodes are connected to the field generator, allowing connection of a total of 8 animals (in 4 dyadic cages).

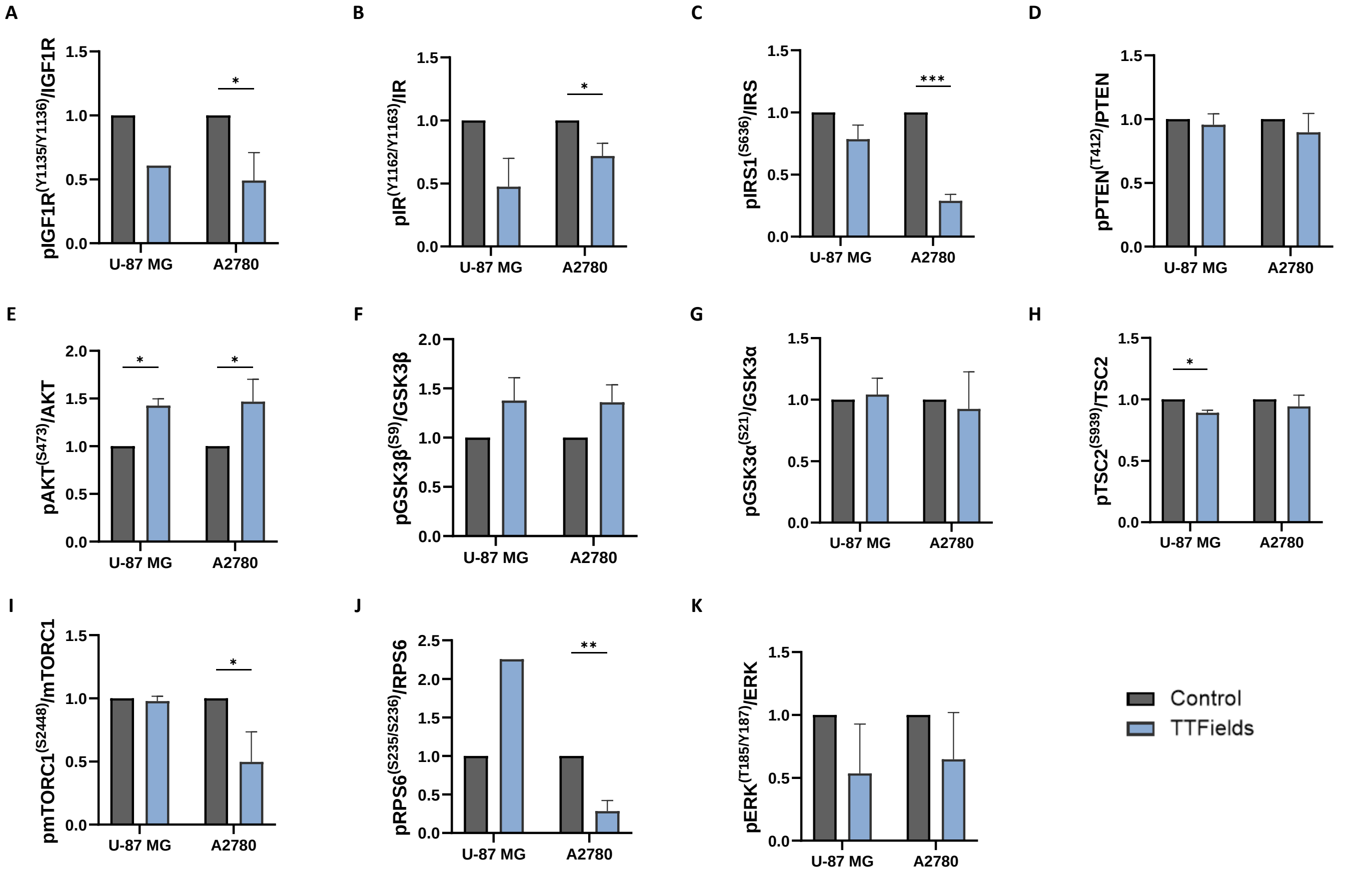

Figure S2. Supporting data for the Luminex assay.

Luminex assay of lysates from control and TTFields-treated (72h) U-87 MG and A2780 cells. Fold change of phosphorylated to total protein ratio relative to the control is presented for: **(A)** IGF1R (Tyr1135/Tyr1136); **(B)** IR (Tyr1162/Tyr1163); **(C)** IRS1 (Ser636); **(D)** PTEN (Thr412); **(E)** AKT (Ser473); **(F)** GSK3β (Ser9); **(G)** GSK3α (Ser21); **(H)** TSC2 (Ser939); **(I)** mTORC1 (Ser2448); **(J)** RPS6 (Ser235/Ser236); and **(K)** ERK (Thr185/Tyr187). Data are presented as mean ± SEM; *p < 0.05; **p < 0.01; and ***p < 0.001; multiple unpaired t-test N ≥ 2.

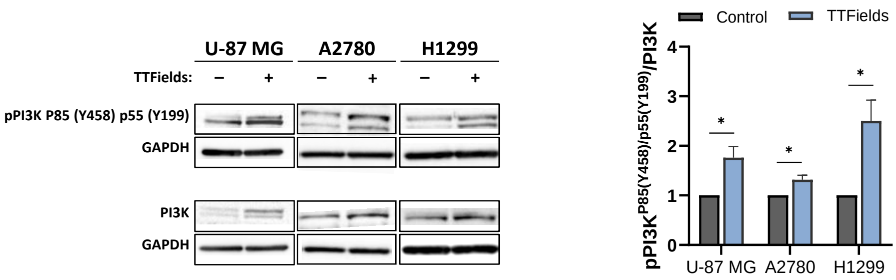

Figure S3. PI3K activation following TTFields application.

Western blot analysis of PI3K, pPI3K (Tyr458 of the PI3K p85 regulatory subunit), and GAPDH in lysates from control and TTFields-treated (72h) U-87 MG, A2780, and H1299 cells. Densitometric analysis of phosphorylation fold change is shown as mean ± SEM. * p < 0.05; multiple unpaired t-tests; *N* = 2.

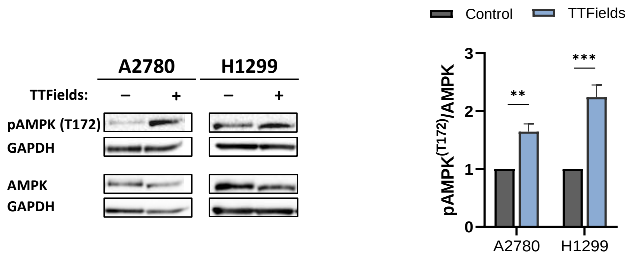

Figure S4. AMPK activation following TTFields application.
Western blot analysis of AMPK, pAMPK (Thr172), and GAPDH in lysates from control and TTFields-treated (72h) A2780 and H1299 cells. Densitometric analysis is shown as mean ± SEM. **p < 0.01; and ***p < 0.001; multiple unpaired t-tests; *N* = 2.

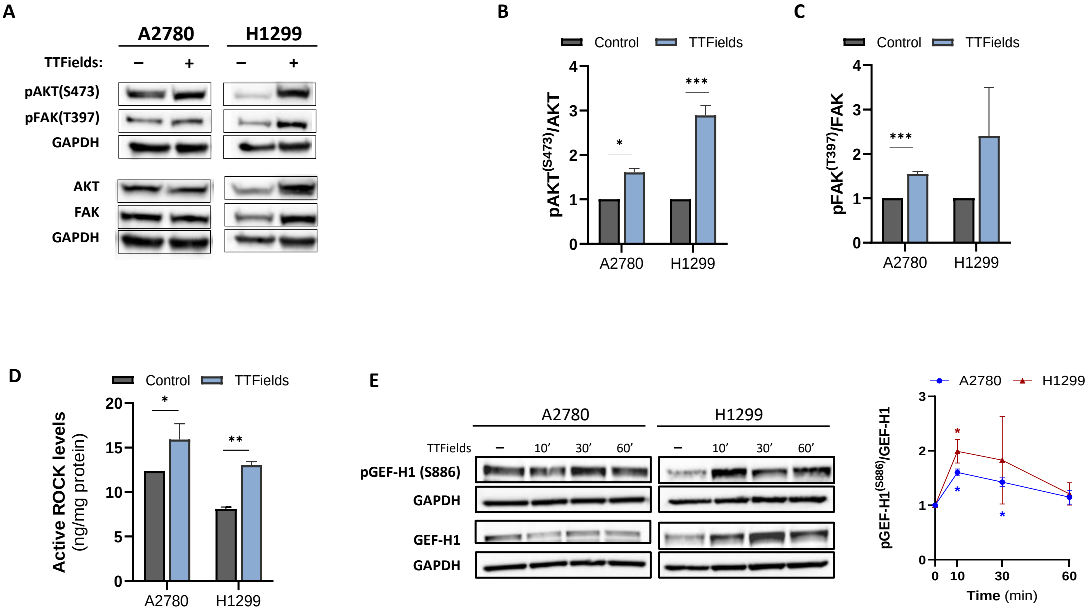

Figure S5. AKT, FAK, ROCK, and GEF-H1 activation following short-term TTFields application at low cell confluency.
(A-C) Western blot analysis of AKT, pAKT (Ser473), FAK, pFAK (Tyr397), and GAPDH in lysates from control and TTFields-treated (10min) A2780 and H1299 cells. Densitometric analysis of phosphorylation fold change is shown as mean ± SEM; *N* = 2. * p < 0.05, ** p < 0.01, and *** p < 0.001; multiple unpaired t-tests. **(D)** ROCK activity from control and TTFields-treated (10 min) A2780 and H1299 cells, shown as mean ± SEM. * p < 0.05, and ** p < 0.01; multiple unpaired t-tests, *N = 2*. **(E)** Western blot analysis of for GEF-H1, pGEF-H1 (Ser886), and GAPDH in lysates from control and TTFields-treated (10, 30 or 60 min) A2780 and H1299 cells. Densitometric analysis is shown as mean ± SEM. * p < 0.05; multiple unpaired t-tests; *N* = 2.

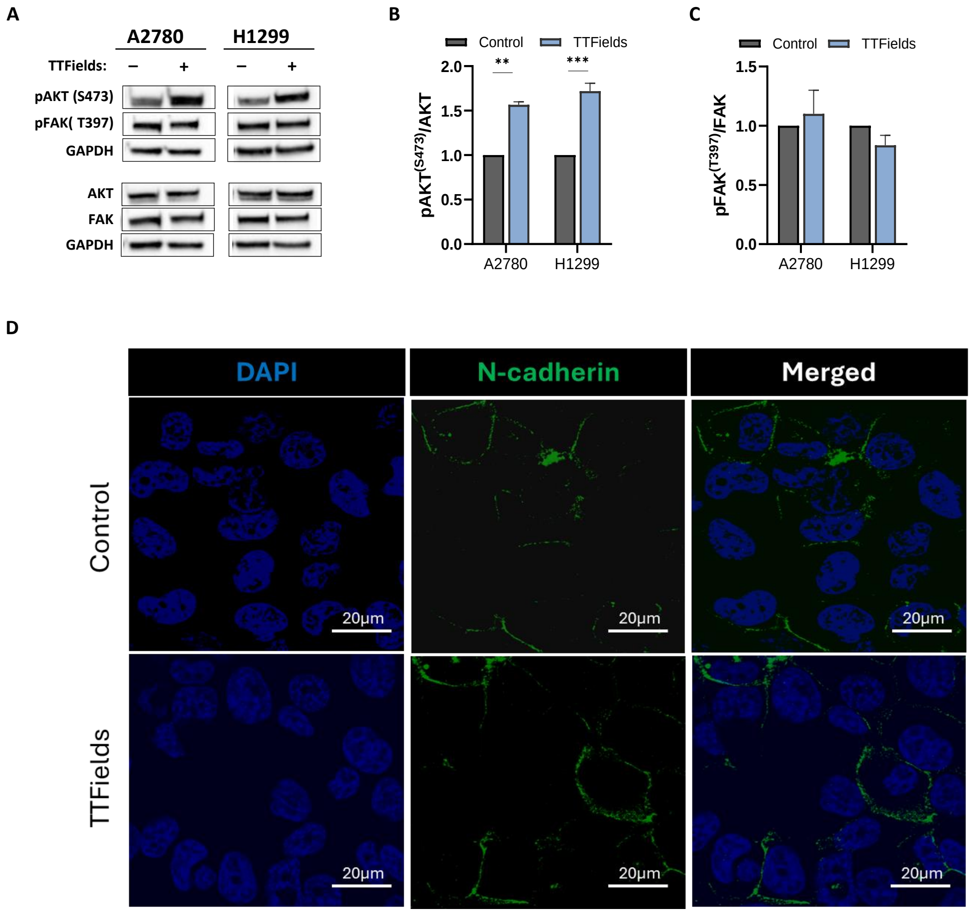

Figure S6. N-cadherin activation and FAK lack of activation following short-term TTFields application at high cell confluency.
(A-C) Western blot analysis of AKT, pAKT (Ser473), FAK, pFAK (Tyr397), and GAPDH in lysates from control and TTFields-treated A2780 and H1299 cells, performed following 2h treatment of 20×10^4 cells/coverslip. Densitometric analysis of phosphorylation fold change is shown as mean ± SEM; $N = 2$. * $p < 0.05$, ** $p < 0.01$, and *** $p < 0.001$; multiple unpaired t-tests. **(D)** Confocal fluorescence microscopy images (x63 magnification) of N-cadherin in the control and TTFields-treated H1299 cells, performed following 2h treatment of 20×10^4 cells/coverslip.

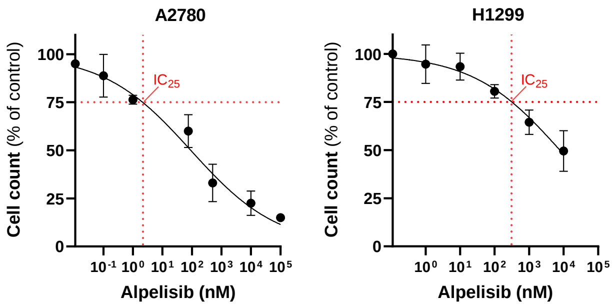

Figure S7. Alpelisib dose-response curves.
Cell count of A2780 and H1299 cells treated (72h) with escalating alpelisib doses. The intersection of the dashed lines indicates the IC_{25}. Values are mean ± SEM. $N = 2$.

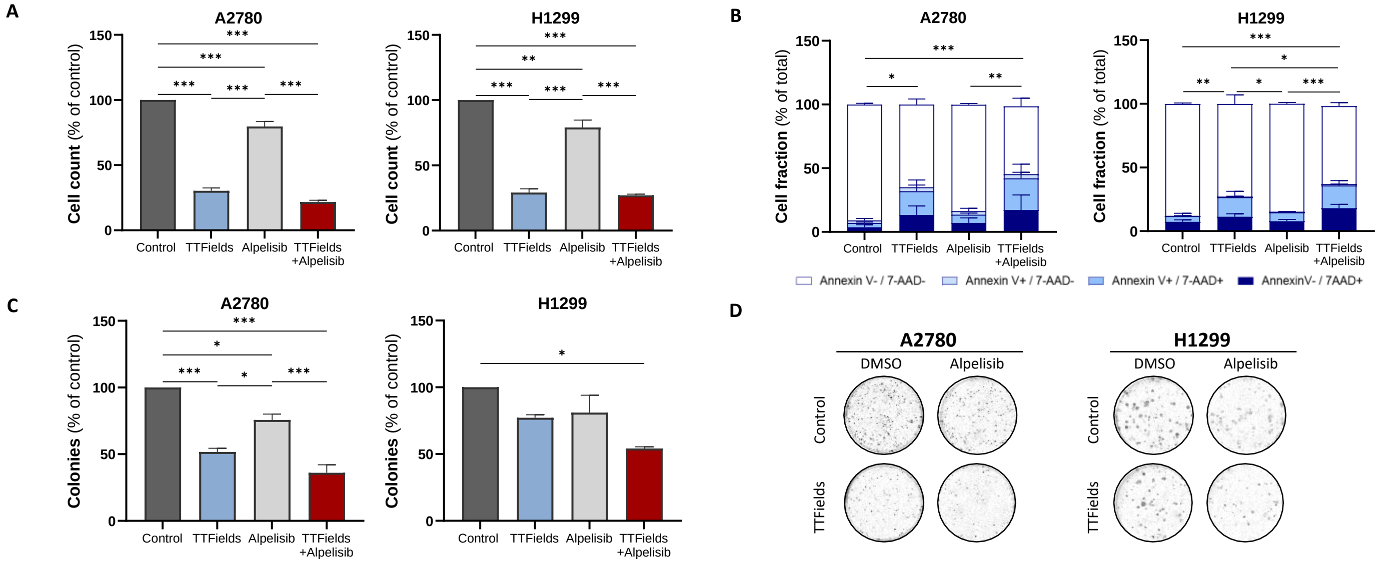

Figure S8. Concomitant treatment with TTFields and alpelisib *in vitro*.
Cell counts (A), apoptosis [Live: Annexin V-/7AAD-; early apoptosis: Annexin V+/7AAD-; late apoptosis: Annexin V+/7AAD+] (B), and clonogenic survival (C-D) of control and TTFields-treated (72h) A2780 and H1299 cells, with or without concomitant alpelisib throughout. Results are shown as mean ± SEM; ; $N = 2$. * $p < 0.05$, ** $p < 0.01$, *** $p < 0.001$, and **** $p < 0.0001$; one-way ANOVA followed by Tukey's post hoc test for cell count and clonogenic survival; two-way ANOVA followed by Tukey's post hoc test for apoptosis, with significance within shown for the live cell fraction.

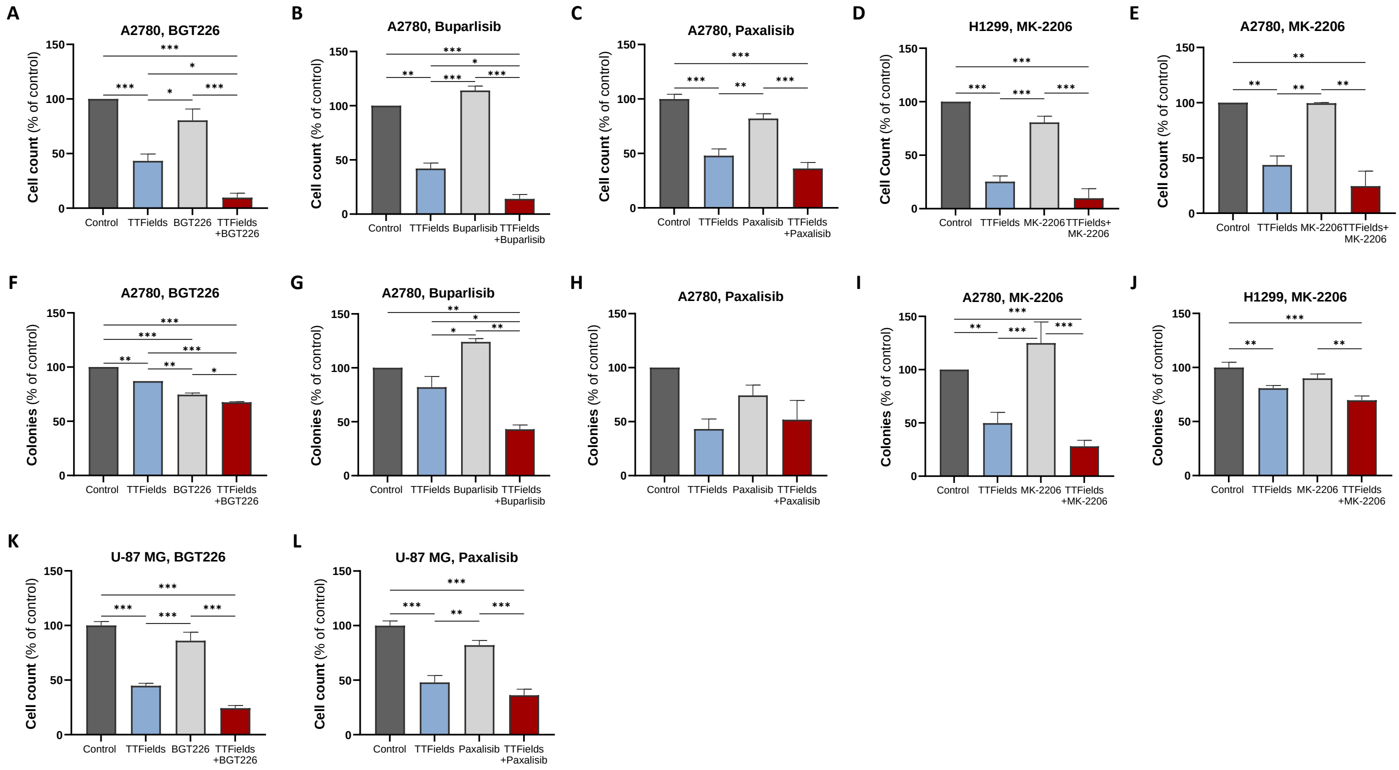

Figure S9. Concomitant treatment with TTFields and PI3K/AKT inhibitors *in vitro*.
Cell counts **(A-E)** and clonogenic survival **(F-J)** of control and TTFields-treated (144h) A2780 **(A-D** and **F-I)** or H1299 cells **(E** and **J)**, with or without concomitant BGT226 (A and F), buparlisib **(B** and **G)**, paxalisib **(C** and **H)**, or MK-2206 **(D, E** and **I, J)** (during the first 72h). Cell counts **(K-L)** of control and TTFields-treated (240h) U-87 MG cells, with or without concomitant BGT226 **(G)**, or paxalisib **(L)** (during the first 72h). Results are shown as mean ± SEM; $N = 2$. * $p < 0.05$, ** $p < 0.01$, and *** $p < 0.001$; one-way ANOVA followed by Tukey's post hoc test for cell count and clonogenic survival.

Figure 1

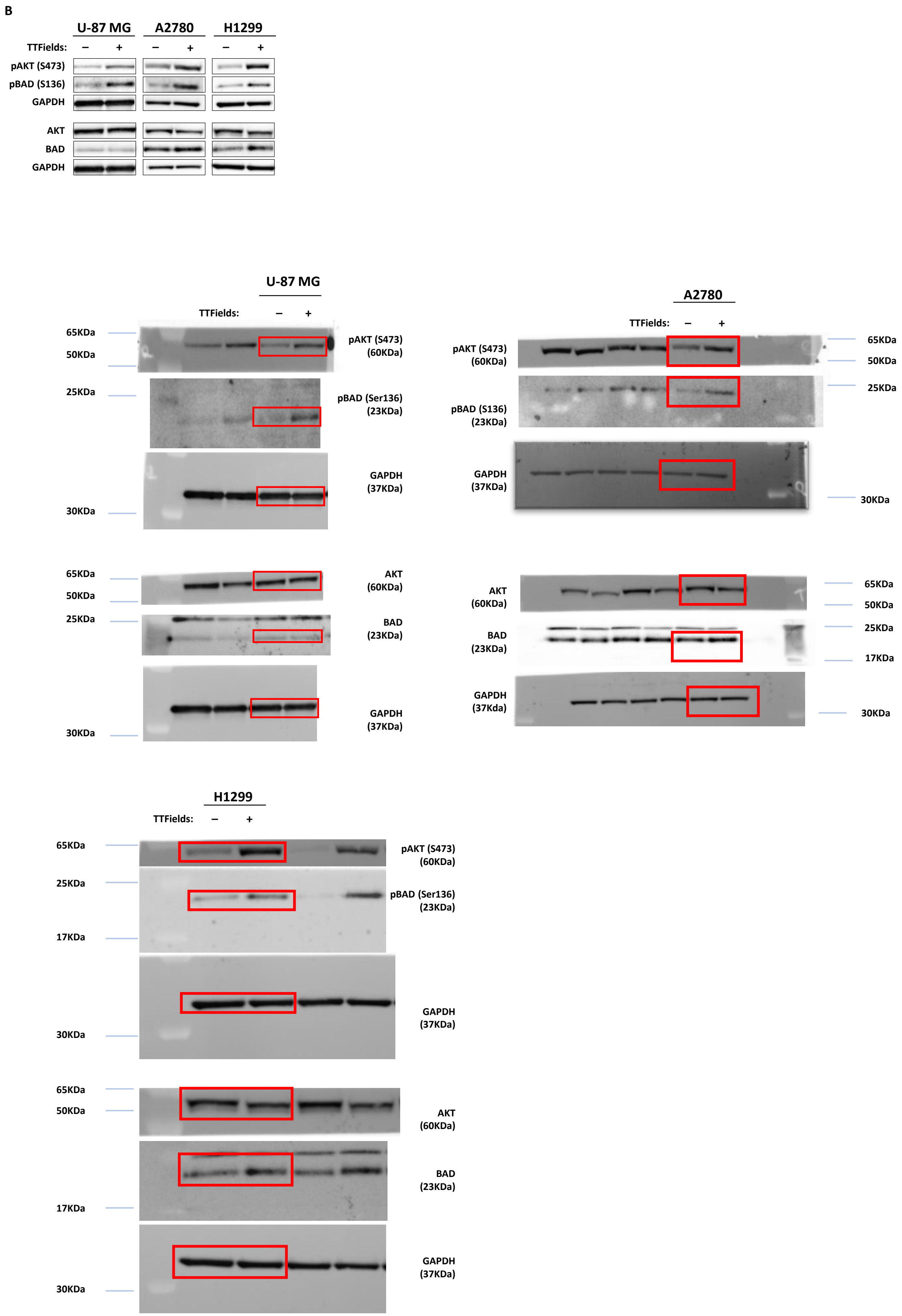
B
U-87 MG
A2780
H1299
TTFields:
–
+
pAKT (S473)
pBAD (S136)
GAPDH
AKT
BAD
GAPDH
U-87 MG
TTFields: – +
65KDa
50KDa
pAKT (S473)
(60KDa)
25KDa
pBAD (Ser136)
(23KDa)
GAPDH
(37KDa)
30KDa
65KDa
50KDa
AKT
(60KDa)
25KDa
BAD
(23KDa)
GAPDH
(37KDa)
30KDa
A2780
TTFields: – +
pAKT (S473)
(60KDa)
65KDa
50KDa
25KDa
pBAD (S136)
(23KDa)
GAPDH
(37KDa)
30KDa
AKT
(60KDa)
65KDa
50KDa
BAD
(23KDa)
25KDa
17KDa
GAPDH
(37Kda)
30KDa
H1299
TTFields: – +
65KDa
pAKT (S473)
(60KDa)
25KDa
pBAD (Ser136)
(23KDa)
17KDa
GAPDH
(37KDa)
30KDa
65KDa
50KDa
AKT
(60KDa)
BAD
(23KDa)
17KDa
GAPDH
(37KDa)
30KDa

Figure 1

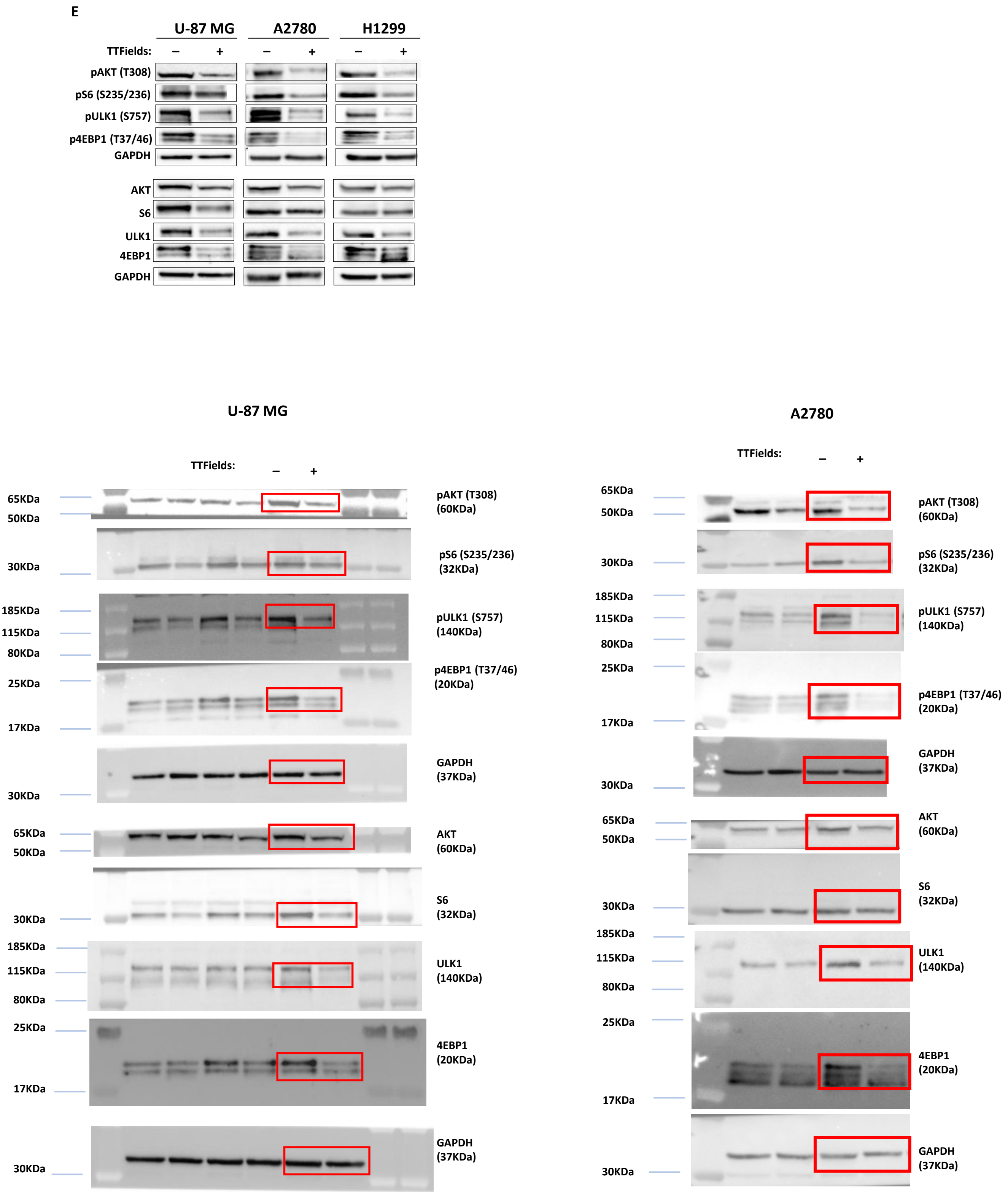

E
U-87 MG
A2780
H1299
TTFields:
–
+
pAKT (T308)
pS6 (S235/236)
pULK1 (S757)
p4EBP1 (T37/46)
GAPDH
AKT
S6
ULK1
4EBP1
GAPDH
U-87 MG
A2780
TTFields:
65KDa
50KDa
30KDa
185KDa
115KDa
80KDa
25KDa
17KDa
pAKT (T308) (60KDa)
pS6 (S235/236) (32KDa)
pULK1 (S757) (140KDa)
p4EBP1 (T37/46) (20KDa)
GAPDH (37KDa)
AKT (60KDa)
S6 (32KDa)
ULK1 (140KDa)
4EBP1 (20KDa)
GAPDH (37KDa)

Figure 1

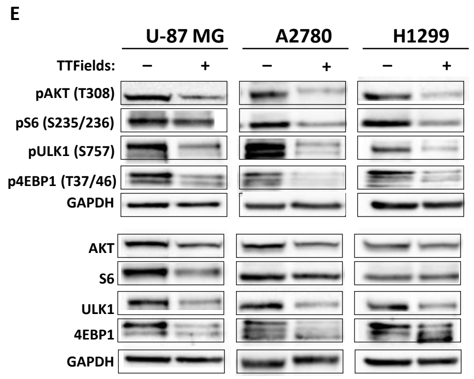

E
U-87 MG
A2780
H1299
TTFields: – + – + – +
pAKT (T308)
pS6 (S235/236)
pULK1 (S757)
p4EBP1 (T37/46)
GAPDH
AKT
S6
ULK1
4EBP1
GAPDH

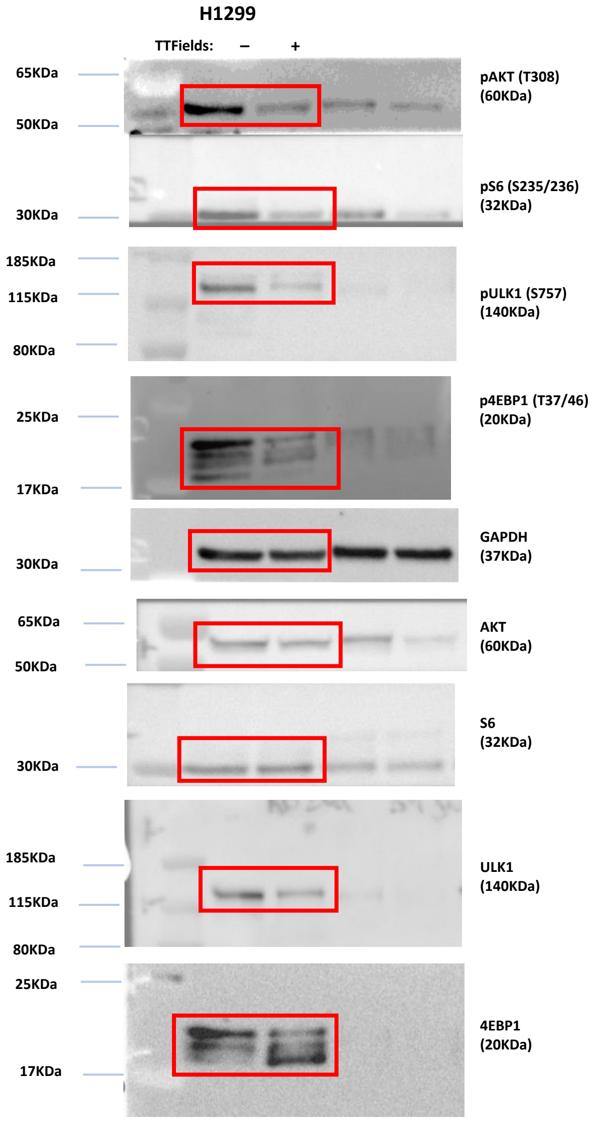

H1299
TTFields: – +
65KDa
50KDa
pAKT (T308)
(60KDa)
30KDa
pS6 (S235/236)
(32KDa)
185KDa
115KDa
80KDa
pULK1 (S757)
(140KDa)
25KDa
17KDa
p4EBP1 (T37/46)
(20KDa)
30KDa
GAPDH
(37KDa)
65KDa
50KDa
AKT
(60KDa)
30KDa
S6
(32KDa)
185KDa
115KDa
80KDa
ULK1
(140KDa)
25KDa
17KDa
4EBP1
(20KDa)

Figure 2

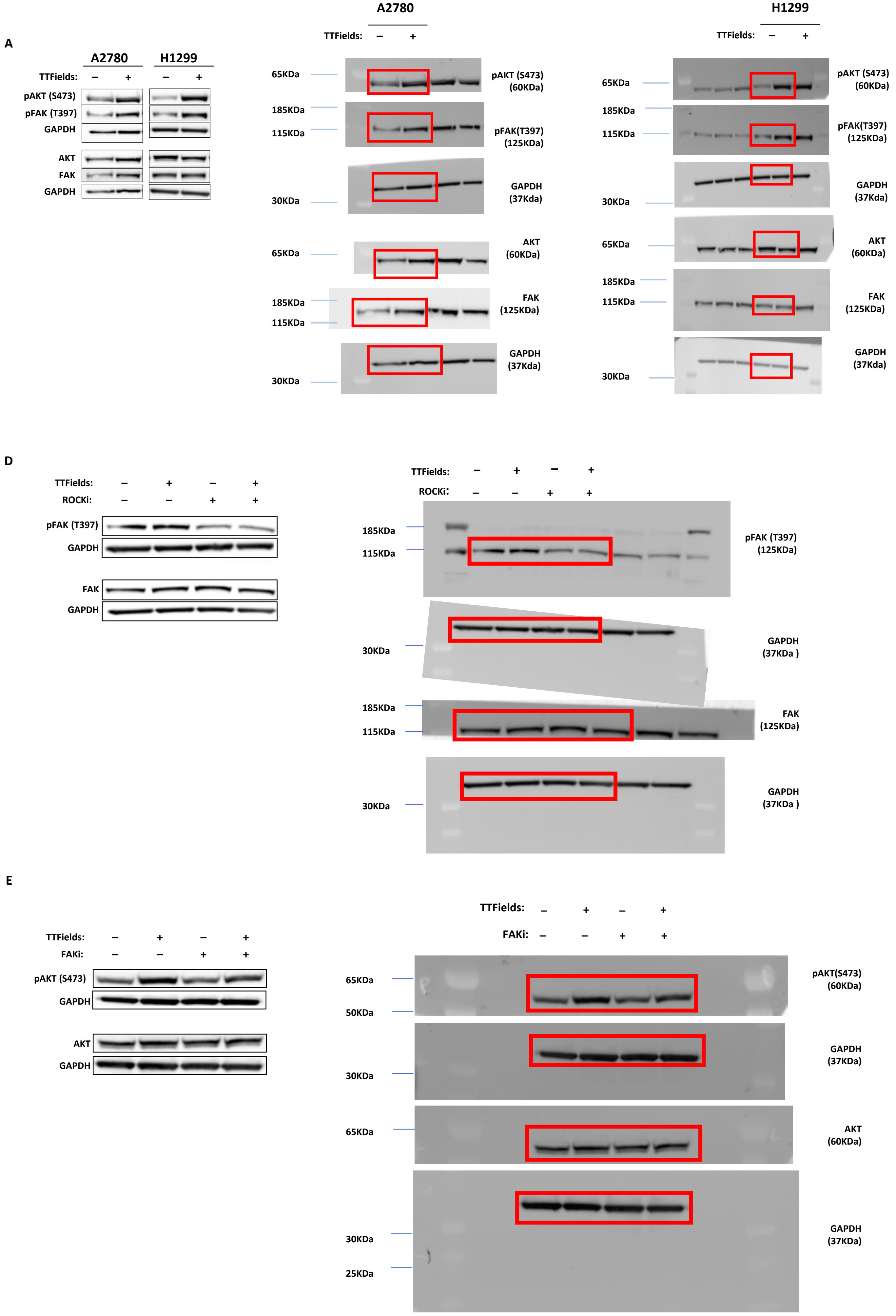

A
A2780
H1299
TTFields: – + – +
pAKT (S473)
pFAK (T397)
GAPDH
AKT
FAK
GAPDH
A2780
TTFields: – +
65KDa
pAKT (S473) (60KDa)
185KDa
115KDa
pFAK(T397) (125KDa)
30KDa
GAPDH (37Kda)
65KDa
AKT (60KDa)
185KDa
115KDa
FAK (125KDa)
30KDa
GAPDH (37Kda)
H1299
TTFields: – +
65KDa
pAKT (S473) (60KDa)
185KDa
115KDa
pFAK(T397) (125KDa)
30KDa
GAPDH (37Kda)
65KDa
AKT (60KDa)
185KDa
115KDa
FAK (125KDa)
30KDa
GAPDH (37Kda)
D
TTFields: – + – +
ROCKi: – – + +
pFAK (T397)
GAPDH
FAK
GAPDH
TTFields: – + – +
ROCKi: – – + +
185KDa
115KDa
pFAK (T397) (125KDa)
30KDa
GAPDH (37KDa)
185KDa
115KDa
FAK (125KDa)
30KDa
GAPDH (37KDa)
E
TTFields: – + – +
FAKi: – – + +
pAKT (S473)
GAPDH
AKT
GAPDH
TTFields: – + – +
FAKi: – – + +
65KDa
50KDa
pAKT(S473) (60KDa)
30KDa
GAPDH (37KDa)
65KDa
AKT (60KDa)
30KDa
25KDa
GAPDH (37KDa)

Figure 2

F

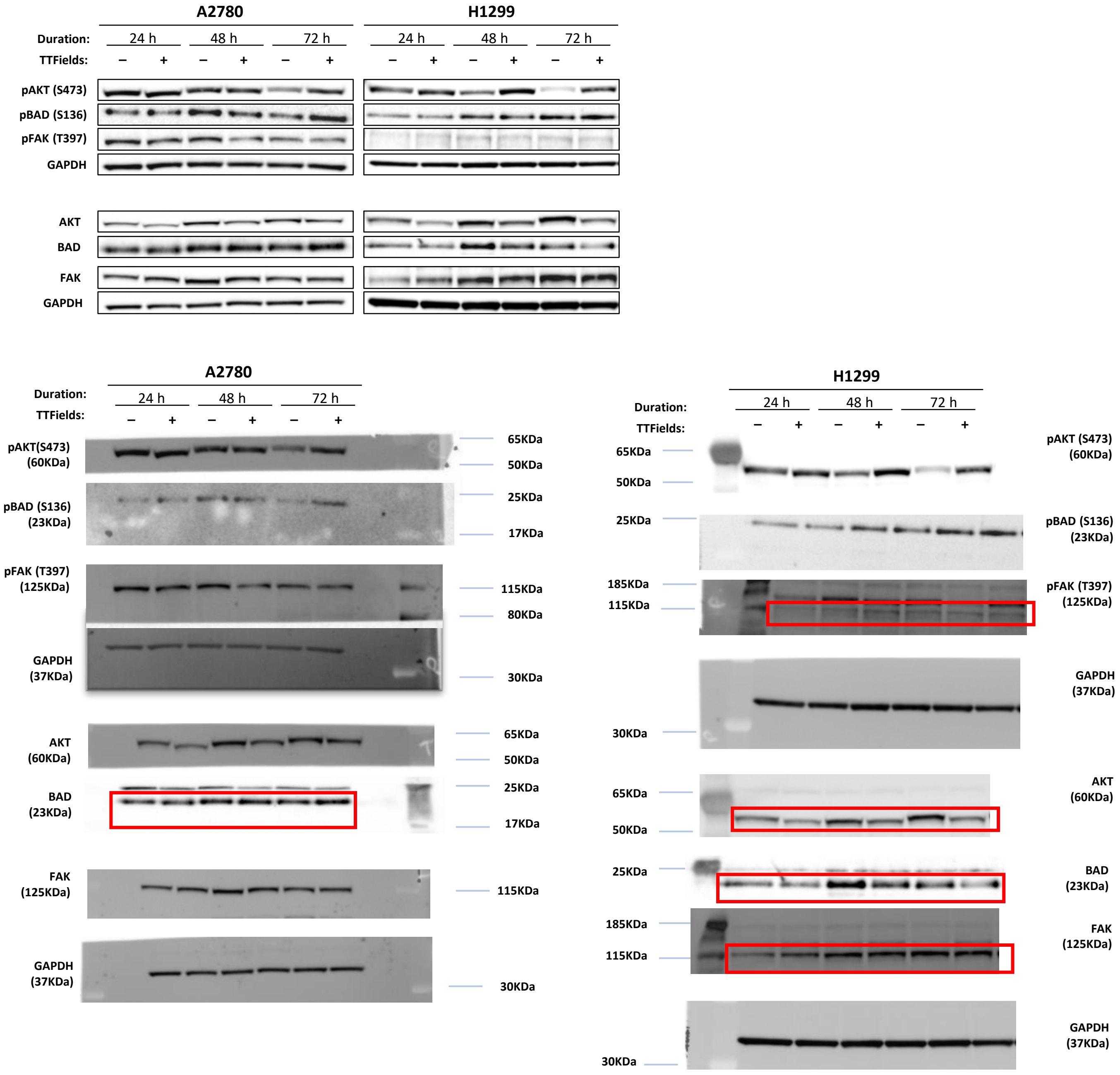

A2780
H1299
Duration:
24 h
48 h
72 h
TTFields:
– + – + – +
pAKT (S473)
pBAD (S136)
pFAK (T397)
GAPDH
AKT
BAD
FAK
GAPDH
pAKT(S473) (60KDa)
pBAD (S136) (23KDa)
pFAK (T397) (125KDa)
GAPDH (37KDa)
AKT (60KDa)
BAD (23KDa)
FAK (125KDa)
GAPDH (37KDa)
185KDa
115KDa
80KDa
65KDa
50KDa
30KDa
25KDa
17KDa

Figure 3

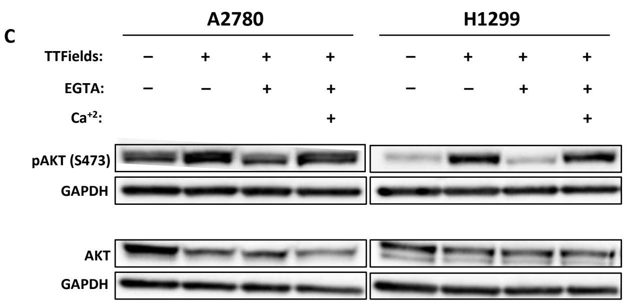
C
A2780
H1299
TTFields:
EGTA:
Ca+2:
pAKT (S473)
GAPDH
AKT
GAPDH

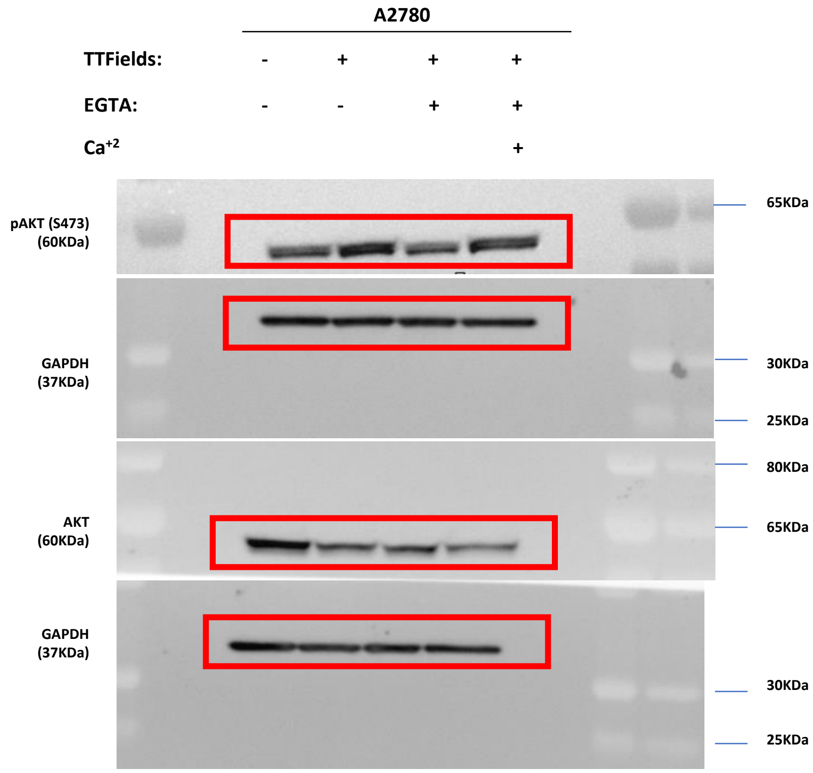
A2780
TTFields:
EGTA:
Ca+2
pAKT (S473)
(60KDa)
GAPDH
(37KDa)
AKT
(60KDa)
GAPDH
(37KDa)
65KDa
30KDa
25KDa
80KDa
65KDa
30KDa
25KDa

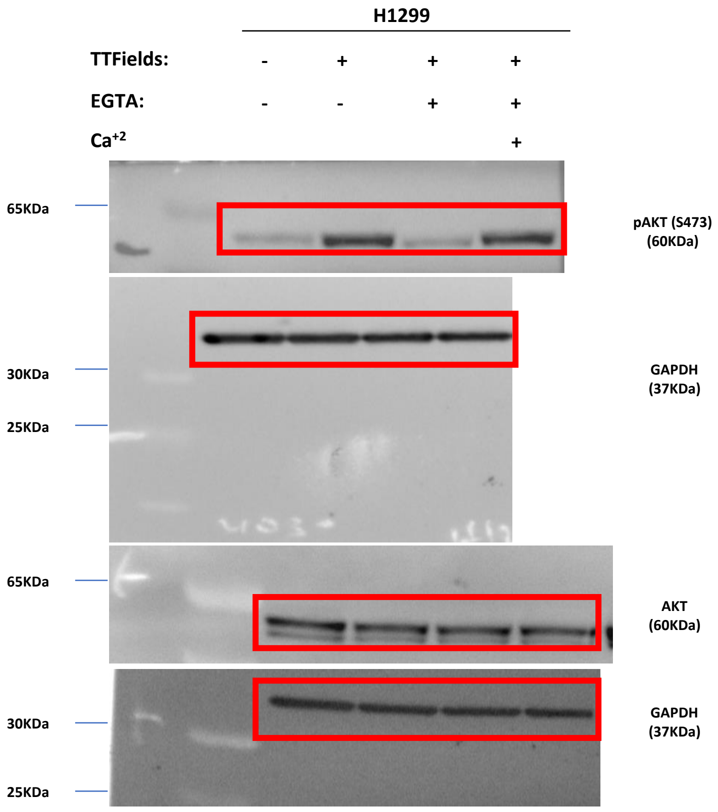
H1299
TTFields:
EGTA:
Ca+2
65KDa
30KDa
25KDa
65KDa
30KDa
25KDa
pAKT (S473)
(60KDa)
GAPDH
(37KDa)
AKT
(60KDa)
GAPDH
(37KDa)

Figure 3

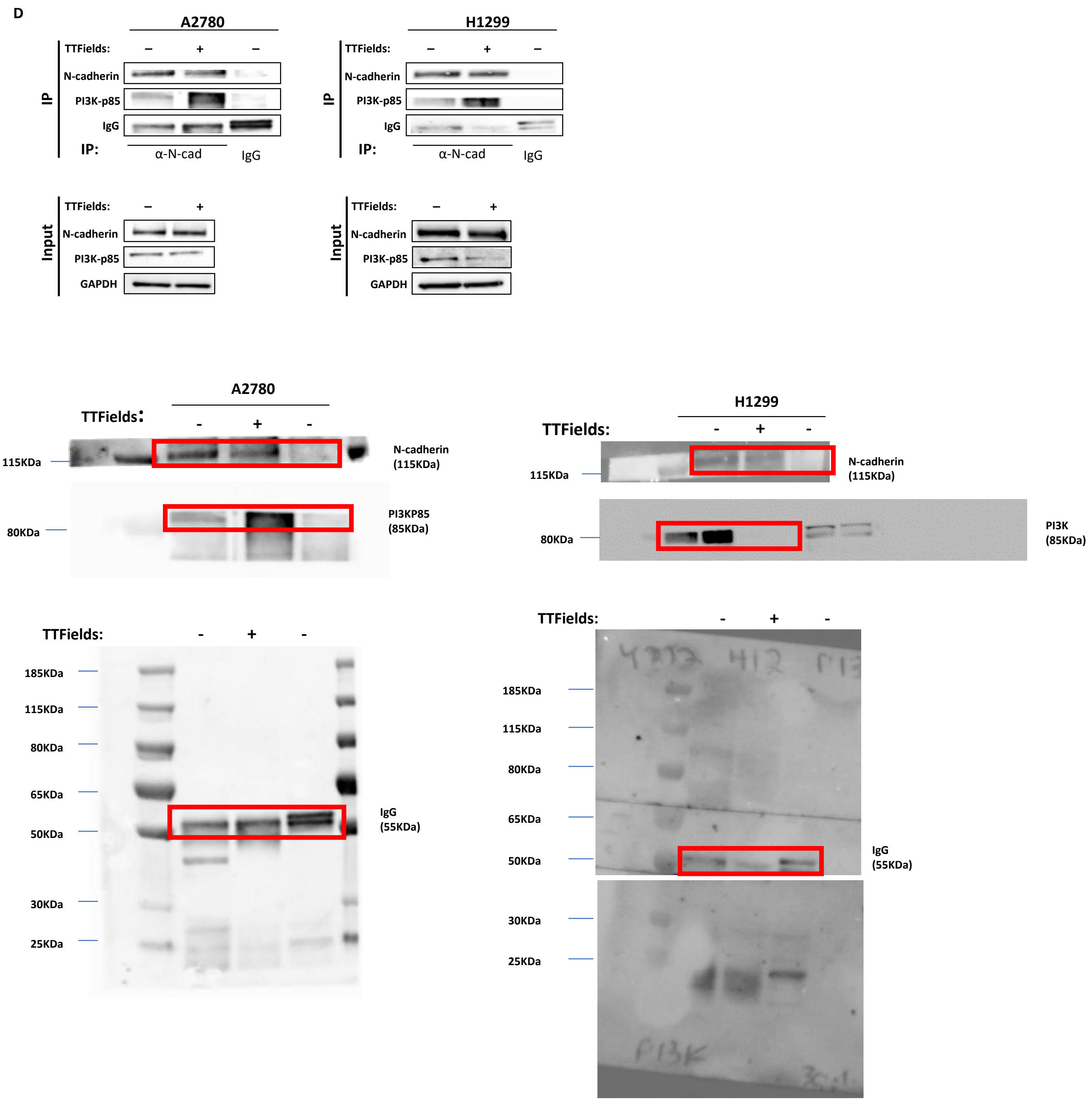
D
A2780
H1299
TTFields:
N-cadherin
PI3K-p85
IgG
IP
IP:
α-N-cad
IgG
Input
GAPDH
115KDa
80KDa
N-cadherin (115KDa)
PI3KP85 (85KDa)
PI3K (85KDa)
185KDa
65KDa
50KDa
30KDa
25KDa
IgG (55KDa)

Figure 3

E

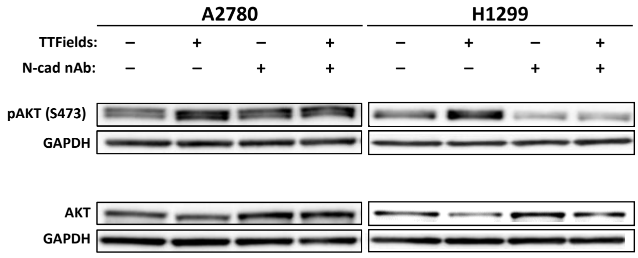
A2780
H1299
TTFields: – + – + – + – +
N-cad nAb: – – + + – – + +
pAKT (S473)
GAPDH
AKT
GAPDH

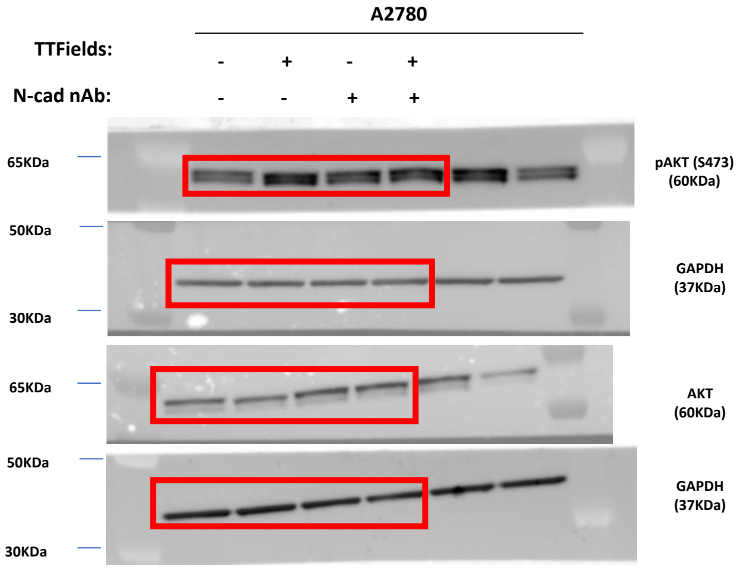
A2780
TTFields: - + - +
N-cad nAb: - - + +
65KDa
50KDa
30KDa
65KDa
50KDa
30KDa
pAKT (S473) (60KDa)
GAPDH (37KDa)
AKT (60KDa)
GAPDH (37KDa)

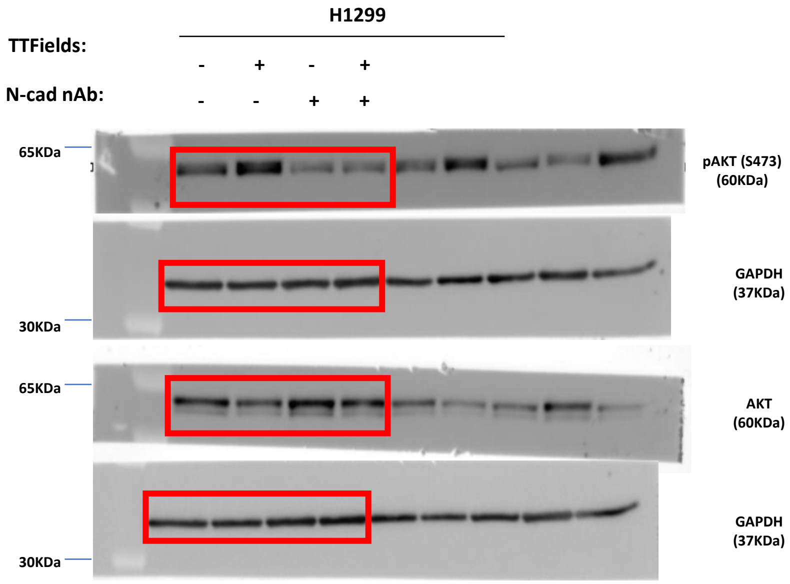
H1299
TTFields: - + - +
N-cad nAb: - - + +
65KDa
30KDa
65KDa
30KDa
pAKT (S473) (60KDa)
GAPDH (37KDa)
AKT (60KDa)
GAPDH (37KDa)

Figure 4

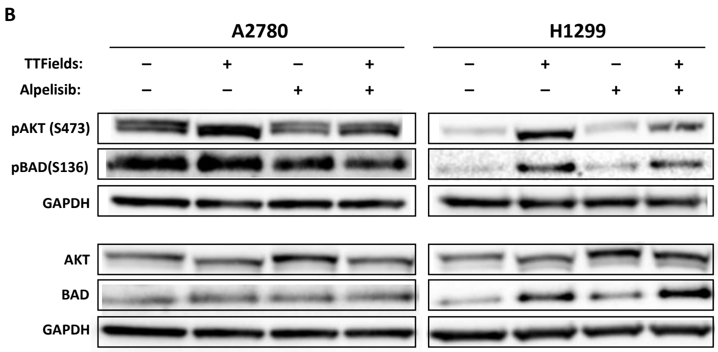
B
A2780
H1299
TTFields: − + − + − + − +
Alpelisib: − − + + − − + +
pAKT (S473)
pBAD(S136)
GAPDH
AKT
BAD
GAPDH

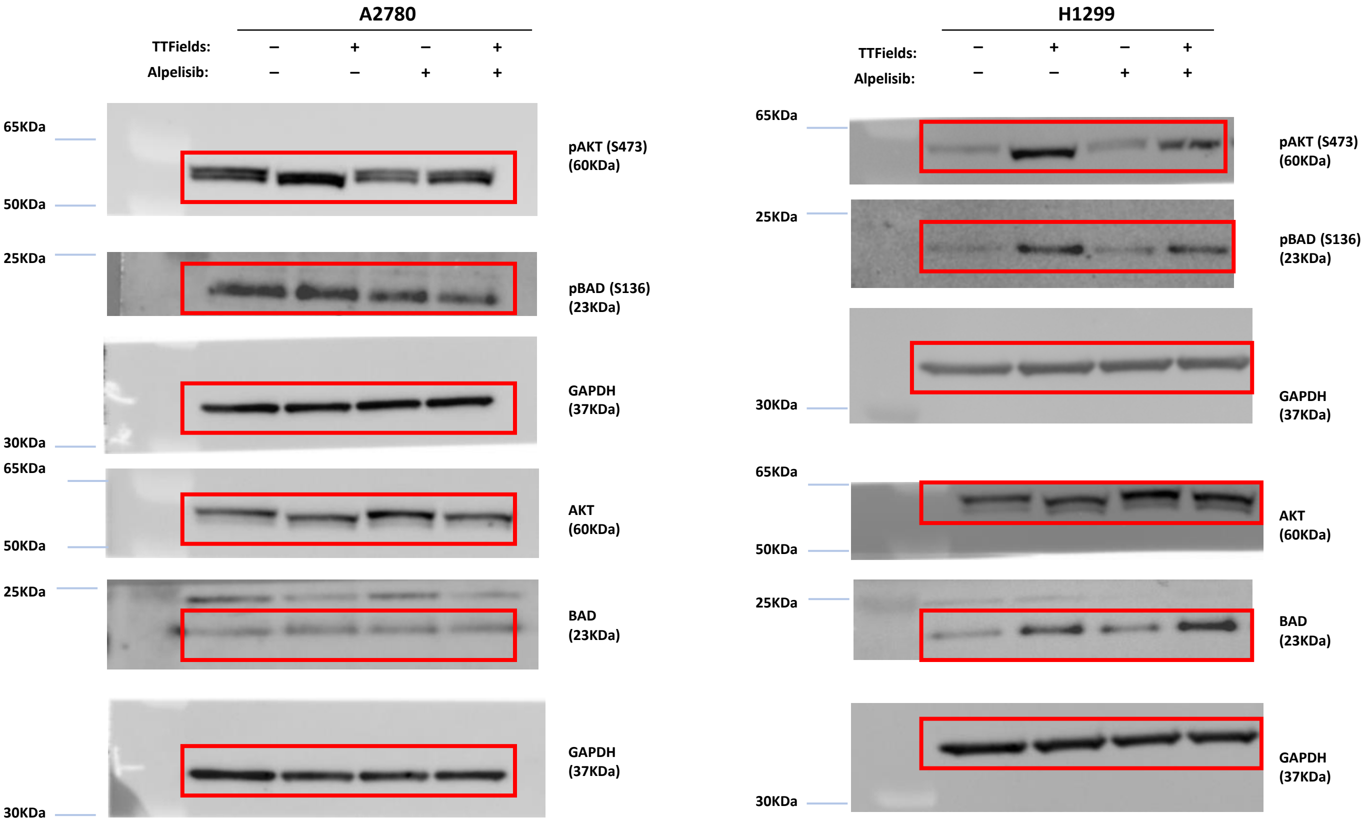
A2780
H1299
TTFields: − + − +
Alpelisib: − − + +
65KDa
50KDa
25KDa
30KDa
pAKT (S473) (60KDa)
pBAD (S136) (23KDa)
GAPDH (37KDa)
AKT (60KDa)
BAD (23KDa)
GAPDH (37KDa)

Figure S3

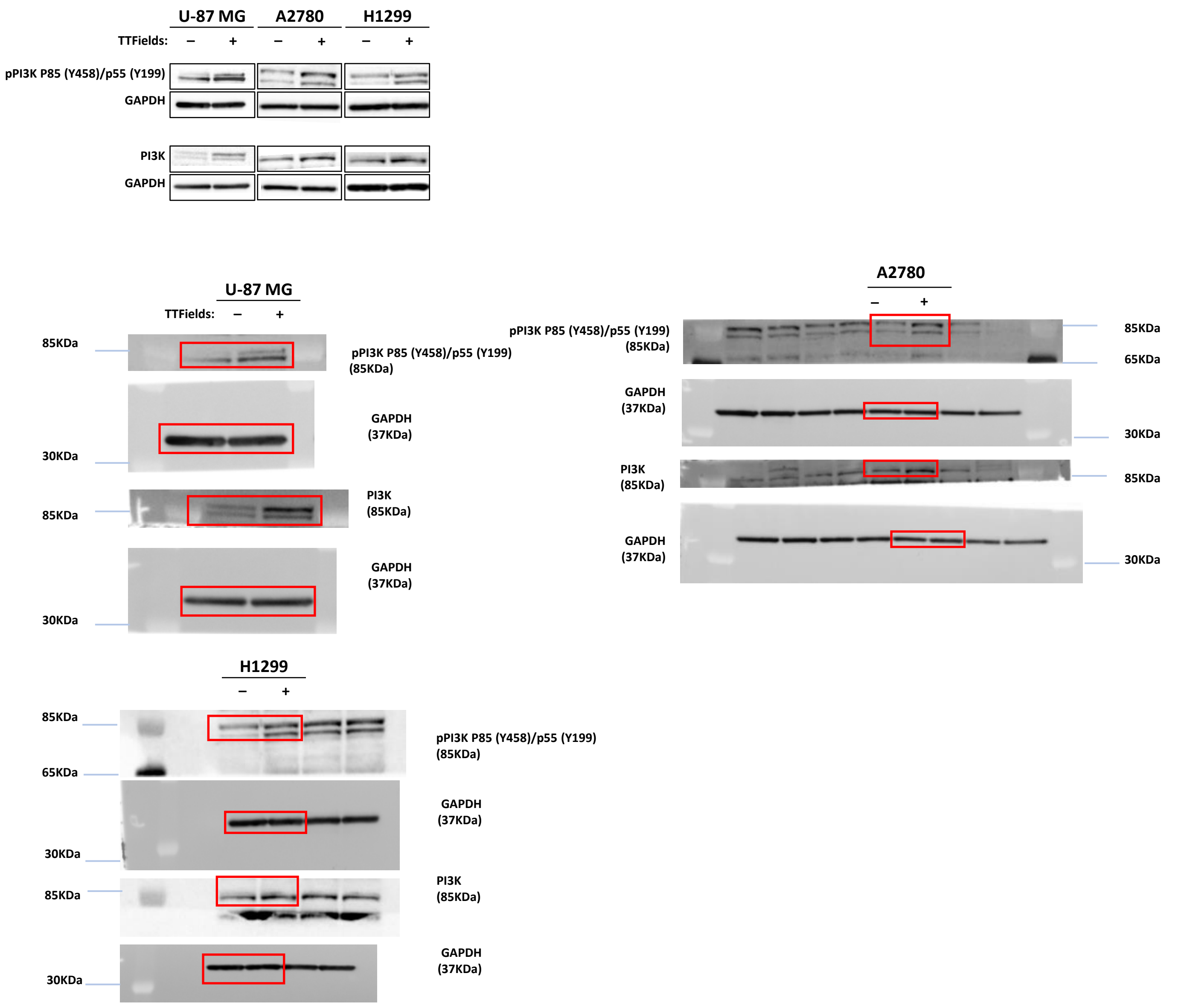

Figure S4

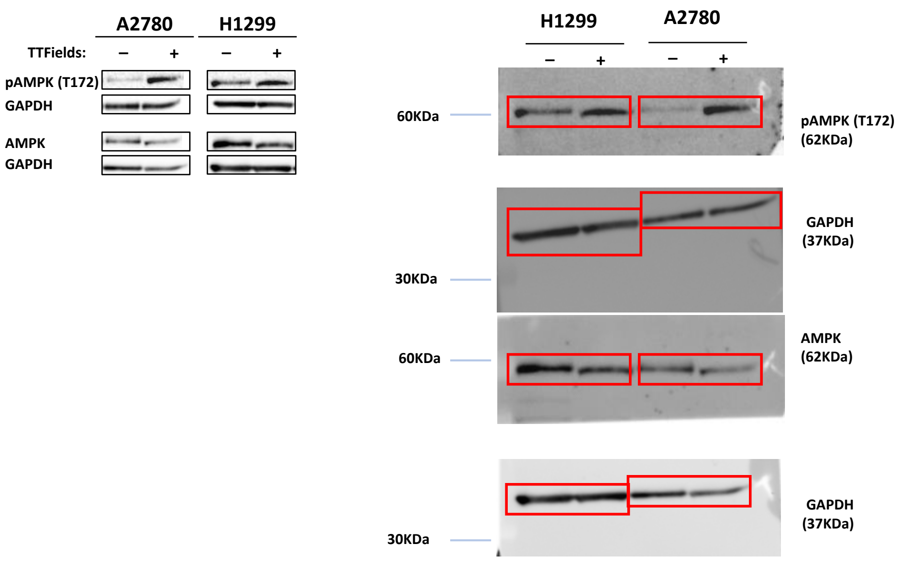

Figure S5

A

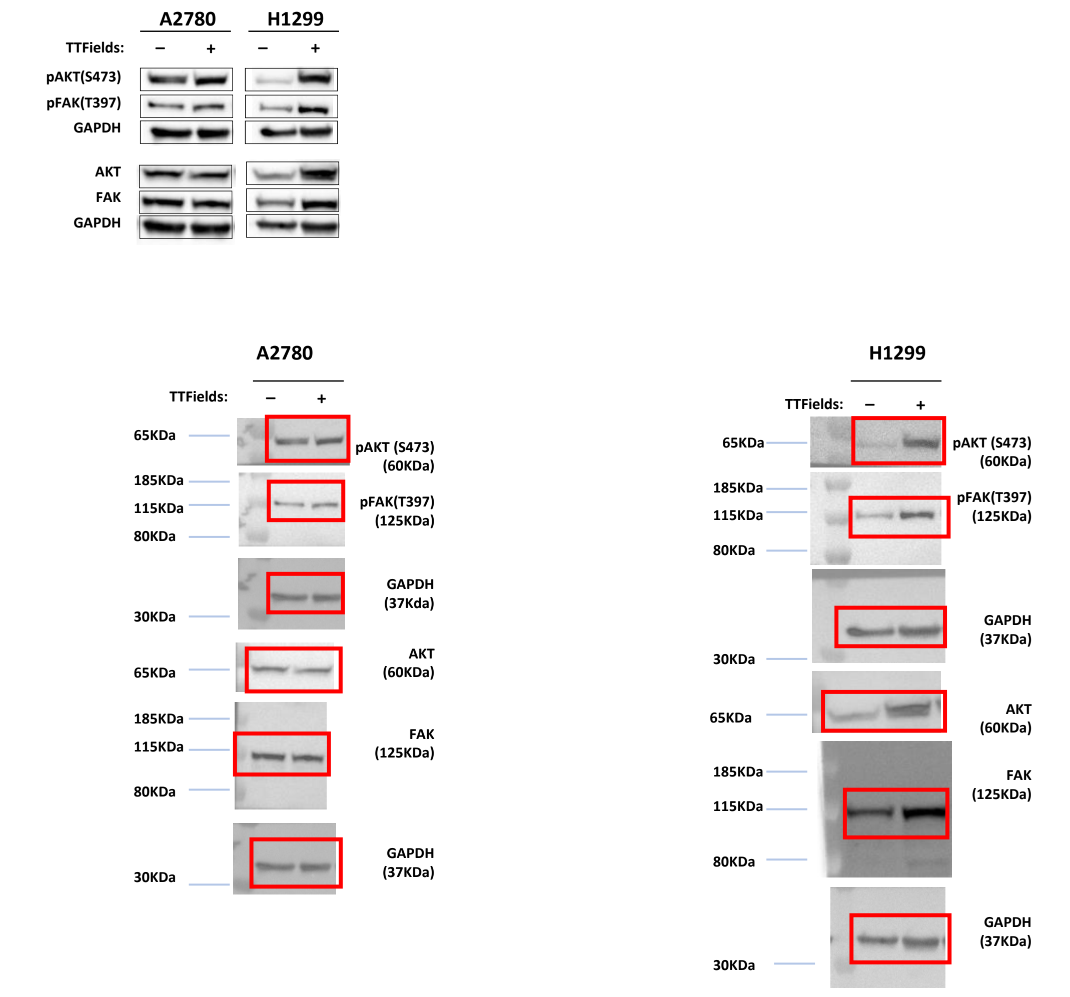

A2780
H1299
TTFields: – + – +
pAKT(S473)
pFAK(T397)
GAPDH
AKT
FAK
GAPDH
A2780
TTFields: – +
65KDa
pAKT (S473) (60KDa)
185KDa
115KDa
80KDa
pFAK(T397) (125KDa)
GAPDH (37Kda)
30KDa
AKT (60KDa)
FAK (125KDa)
GAPDH (37KDa)
H1299
TTFields: – +
pAKT (S473) (60KDa)
pFAK(T397) (125KDa)
GAPDH (37KDa)
AKT (60KDa)
FAK (125KDa)
GAPDH (37KDa)

D

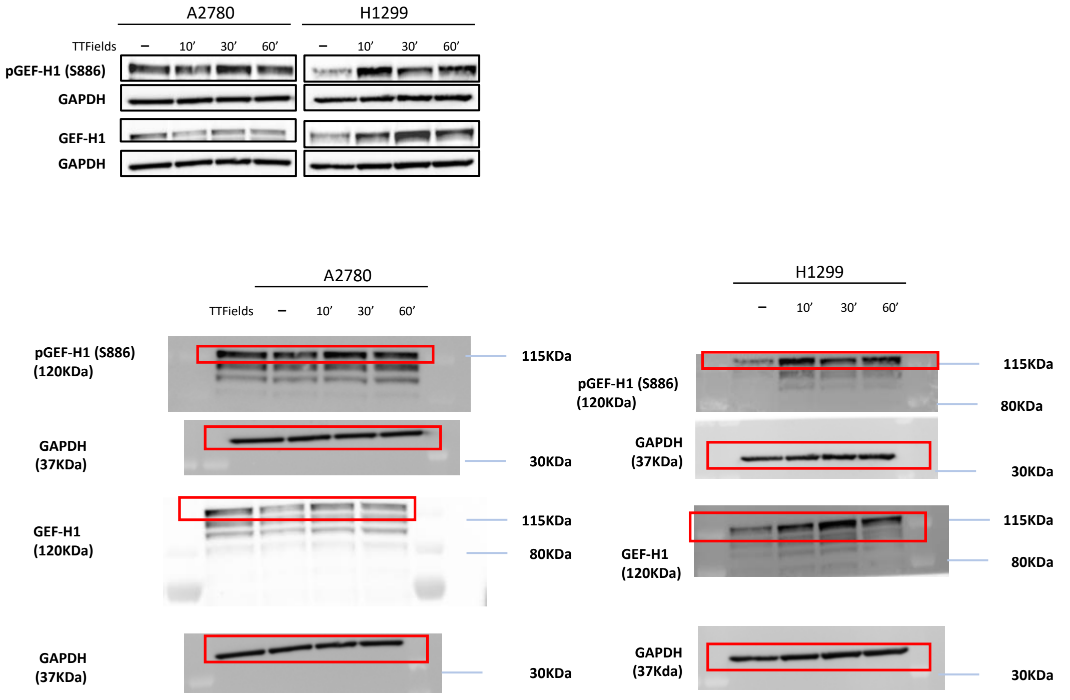

A2780
H1299
TTFields – 10' 30' 60' – 10' 30' 60'
pGEF-H1 (S886)
GAPDH
GEF-H1
GAPDH
A2780
TTFields – 10' 30' 60'
pGEF-H1 (S886) (120KDa)
115KDa
GAPDH (37KDa)
30KDa
GEF-H1 (120KDa)
80KDa
H1299
– 10' 30' 60'
GAPDH (37Kda)
